# Apparent diffusion coefficient measurements in the differentiation between benign and malignant lesions: a systematic review

**DOI:** 10.1007/s13244-012-0175-y

**Published:** 2012-06-07

**Authors:** M. A. Vermoolen, T. C. Kwee, R. A. J. Nievelstein

**Affiliations:** Department of Radiology, University Medical Center, Heidelberglaan 100 HP. E.01.132, PO Box 85500, 3508 GA Utrecht, The Netherlands

**Keywords:** Apparent diffusion coefficient, Diffusion-weighted imaging, Benign, Malignant, Tumour

## Abstract

**Objectives:**

To systematically review the value of apparent diffusion coefficient (ADC) measurement in the differentiation between benign and malignant lesions.

**Methods:**

A systematic search of the Medline/Pubmed and Embase databases revealed 109 relevant studies. Quality of these articles was assessed using the Quality Assessment of the Studies of Diagnostic Accuracy Included in Systematic Reviews (QUADAS) criteria. Reported ADC values of benign and malignant lesions were compared per organ.

**Results:**

The mean quality score of the reviewed articles was 50%. Comparison of ADC values showed marked variation among studies and between benign and malignant lesions in various organs. In several organs, such as breast, liver, and uterus, ADC values discriminated well between benign and malignant lesions. In other organs, such as the salivary glands, thyroid, and pancreas, ADCs were not significantly different between benign and malignant lesions.

**Conclusion:**

The potential utility of ADC measurement for the characterisation of tumours differs per organ. Future well-designed studies are required before ADC measurements can be recommended for the differentiation of benign and malignant lesions. These future studies should use standardised acquisition protocols and provide complete reporting of study methods, to facilitate comparison of results and clinical implementation of ADC measurement for tumour characterisation.

**Electronic supplementary material:**

The online version of this article (doi:10.1007/s13244-012-0175-y) contains supplementary material, which is available to authorized users.

## Introduction

Over the past two decades, magnetic resonance (MR) imaging (MRI) has proven to be a valuable diagnostic tool in oncology [1–4]. Rapid improvements in MRI techniques have resulted in MR images with excellent spatial resolution and soft tissue contrast, which contribute to the differentiation of suspected tumours. However, using conventional MRI sequences, difficulty in differentiating benign from malignant lesions may arise when malignant and benign lesions share certain morphologic and contrast-enhancement characteristics. In these cases, diffusion-weighted MR imaging (DWI) might be of value in tumour assessment, as it has the ability to provide tissue contrast based on molecular diffusion [5]. Since the 1990s, DWI using single-shot echo-planar imaging (EPI) has been successfully applied in the field of neuroradiology. It is particularly valuable in the assessment of acute cerebral ischemia [6, 7]. Initially, DWI in other than intracranial sites did not yield sufficient image quality due to susceptibility artefacts and motion artefacts. More recently, technical advances in MRI, such as the development of parallel imaging, high gradient amplitudes, and multichannel coils, have enabled the performance of DWI in the body. These developments have initiated the investigation of applicability of DWI for tumour characterisation, both intra- and extracranially. Diffusion-weighted images can be assessed in two ways, qualitatively, by visual assessment of signal intensity, and quantitatively, by measurement of the apparent diffusion coefficient (ADC). The ADC value quantifies water proton motion, which in biological tissues is a combination of true water diffusion and capillary perfusion. The ADC value can theoretically be used to characterise tissues, as the degree of diffusion is correlated to cellular density and extracellular space volume [8, 9]. Malignant tumours are reported to have a high cellular density and low extracellular space volume, which is associated with impeded water proton diffusion and low ADC values. In contrast, various benign lesions are characterised by an increased amount of extracellular matrix with minimal increase of cellular density, which may result in higher ADCs [10, 11]. This hypothesis has been investigated for various types of lesions throughout the body. However, because of the large number of studies on this subject with sometimes conflicting results, the utility of ADC measurements in the characterisation of lesions remains unclear. The aim of this study was therefore to systematically review the current literature on the value of ADC measurement in the differentiation between benign and malignant lesions throughout the entire body.

## Materials and methods

### Search strategy

**Fig. 1:**
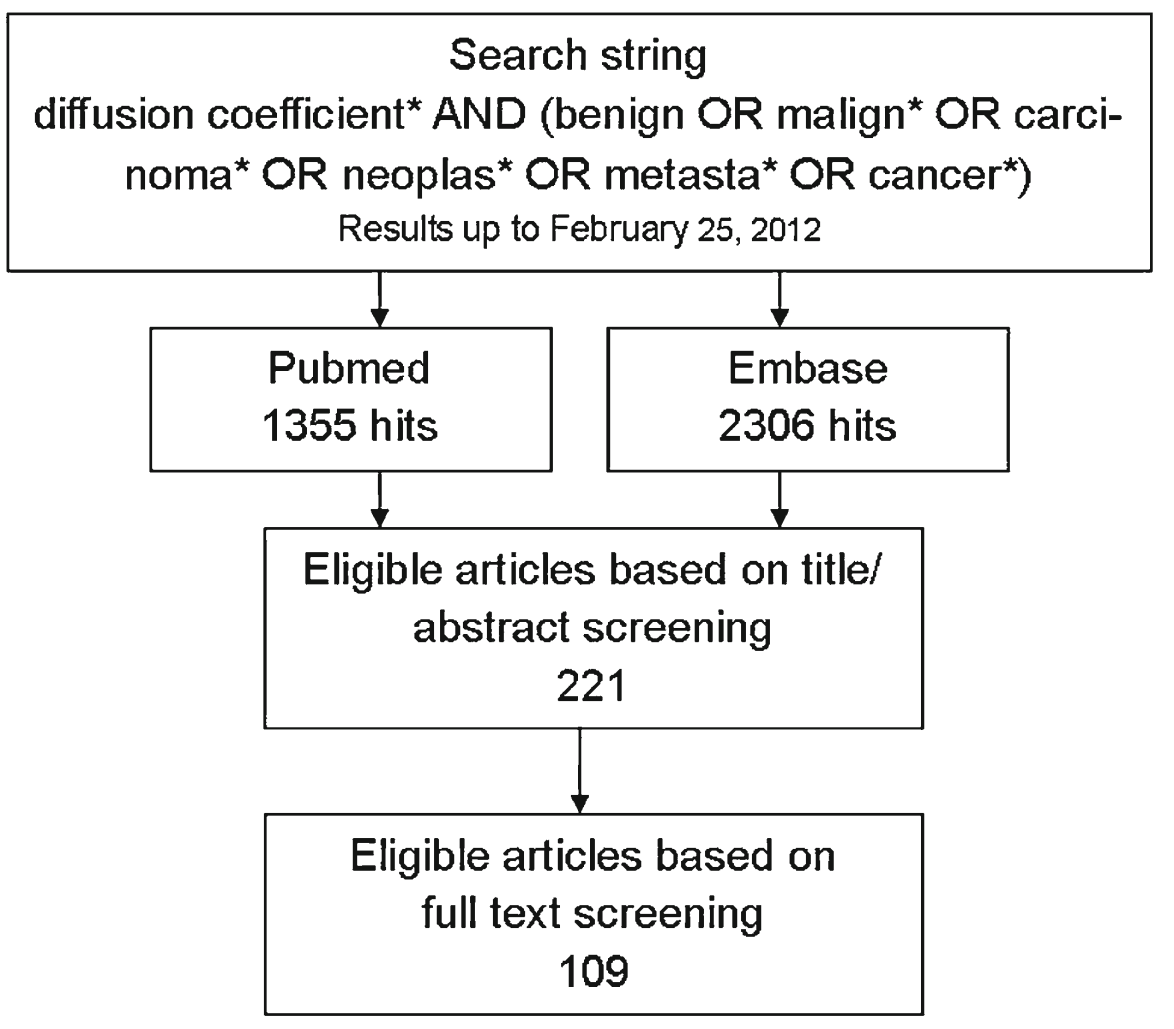
Flow chart of systematic literature search

A systematic literature review was conducted to identify articles on ADC measurements for differentiating benign and malignant lesions (Fig. 1). We performed an electronic search using the Medline/Pubmed and Embase databases. The search string is noted in the flowchart (Fig. 1). No beginning date limit was used and the search was updated until February 25, 2012. No language restriction was applied. Inclusion and exclusion criteria were postulated in consensus by the three authors (M.V./T.K./R.N.) and were applied to the title/abstract and full text screening (Table 1). One researcher (M.V.) screened the titles and abstracts of the search results and selected 221 eligible articles. Full texts were available for 186 of these 221 articles and were screened by one researcher (M.V.). One hundred and nine articles were found to meet the inclusion criteria and were reviewed. The remaining articles were excluded for various reasons, such as lack of histopathological reference standard, the use of ADC ratios instead of true ADC values or describing only the ADC values per histopathological entity, instead of comparing mean ADC values of malignant and benign lesions as a group. Finally, we screened the references of the included articles to find relevant articles that may have been missed in our search, which did not reveal any additional eligible articles. Studies investigating the value of ADC measurements in differentiating benign from malignant prostatic lesions were excluded, because the most important clinical role of DWI with ADC mapping in the prostate is cancer detection and characterisation. In addition, we did not review studies on differentiation of malignant and benign lymphadenopathy, as this subject lies beyond the scope of this paper.

**Table 1:** Inclusion and exclusion criteria

Inclusion criteria	
1	Human, in vivo studies
2	Any language
3	Differentiation between benign and malignant lesions in any organ
4	ADC measurements used to differentiate benign from malignant lesions
5	It was possible to classify ADC measurements into a group of benign and a group of malignant lesions
6	Histological examination used as reference standard
7	Diffusion-weighted MRI with ADC measurements performed prior to any treatment
8	Absolute outcome measures (mean ADCs) can be obtained from article
9	Total sample size of at least 20 lesions
10	MR ≥ 1 Tesla
11	All ages
Exclusion criteria	
1	Therapeutic or prognostic studies
2	Reviews, meta-analyses, editorials, case reports
3	Studies that focus solely on ADC values of (pathologic) lymph nodes or vertebral fractures

*ADC* Apparent diffusion coefficient

### Quality assessment

All relevant papers were assessed for quality using the Quality Assessment of the Studies of Diagnostic Accuracy Included in Systematic Reviews (QUADAS) criteria [12]. We modified this 14-item instrument for optimum applicability to this review. The complete list of criteria is presented in Table 2. One reader (M.V.) assigned positive or negative scores to these criteria for all of the eligible articles. If insufficient information was provided the item was given a negative score. Total quality scores were expressed as a percentage of the maximum score. Quality scores of 0–39% were rated as poor, 40–70% as moderate, and 70–100% as good.

**Table 2:** Adjusted QUADAS^a^ tool for quality assessment

Quality item	Positive score
1. Was the spectrum of patients representative of the patients who will receive the test in practice?	Patients with lesions detected at conventional imaging (e.g., CT, US, or anatomical MRI). Conventional imaging could not assess whether those lesions were benign or malignant
2. Were selection criteria clearly described?	It was clear how patients were selected for inclusion
3. Is the reference standard likely to correctly classify the target condition?	Histological examination was used as a reference standard
4. Was the time period between histological assessment and DWI short enough to be reasonably sure that the target condition did not change between the two tests?	Histological assessment was performed within 2 weeks after DWI
5. Did the whole sample or a random selection of the sample receive verification using a reference standard of diagnosis?	All patients, or a random sample of patients, received histological examination
6. Did patients receive the same reference standard regardless of the index test result?	Patients received histological assessment regardless of ADC measured
7. Was the execution of the index test described in sufficient detail to permit replication of the test?	All of the following MRI parameters are described: field strength, coil type, sequence type, applied *b*-values, BH/RT/FB, and direction(s) of applied diffusion gradients
8. Was the execution of the reference test described in sufficient detail to permit replication?	Description of the following points: means of harvesting histological material (biopsy or surgery) given, interpreter of histological assessment mentioned
9. Were the index test results interpreted without knowledge of the results of the reference standard?	DWI was interpreted without knowledge of the histological assessment findings
10. Were the reference standard results interpreted without knowledge of the results of the index test?	Histological assessment was interpreted without knowledge of the DWI findings
11. Were the same clinical data available when test results were interpreted as would be available when the test is used in practice?	Clinical data were available to the interpreter(s) of the DWI
12. Were withdrawals from the study explained?	Withdrawals from the study after inclusion were explained

*DWI* Diffusion-weighted imaging, *ADC* apparent diffusion coefficient, *BH* breath-hold, *RT* respiratory triggering, *FB* free breathing

^a^Adapted from [12]

### Data presentation

Reported ADC values of the reviewed studies are presented per organ or body region. The reported ADC values (mean ± standard deviation) of benign and malignant tumours in each organ will be discussed and compared and are represented graphically in the accompanying figures. We did not perform meta-analyses as the substantial variation in study characteristics and applied diffusion-weighted imaging parameters of the reviewed studies prevented meaningful pooling of the data. We aimed to give a broad overview of the literature on differentiation between benign and malignant tumours.

## Results

We identified 109 articles that described and compared mean ADC values for malignant and benign tumours in various body regions, of which 14 were intracranial and 95 extracranial. The included extracranial regions were salivary glands (6), thyroid (6), breast (24), lung (2), liver (14), gallbladder (1), pancreas (9), kidney (8), adrenal gland (4), uterus (8), ovaries (7), and soft tissue (6). Study design was prospective in 38 studies, retrospective in 39, and unreported in 32 out of 109 studies. Most studies (98 out of 109) used echo planar imaging (EPI) pulse sequences for diffusion-weighted imaging, and 64 out of 109 studies reported using diffusion gradients in three orthogonal directions (along the *x*, *y*, and *z* axes).

### Study quality

**Fig. 2:**
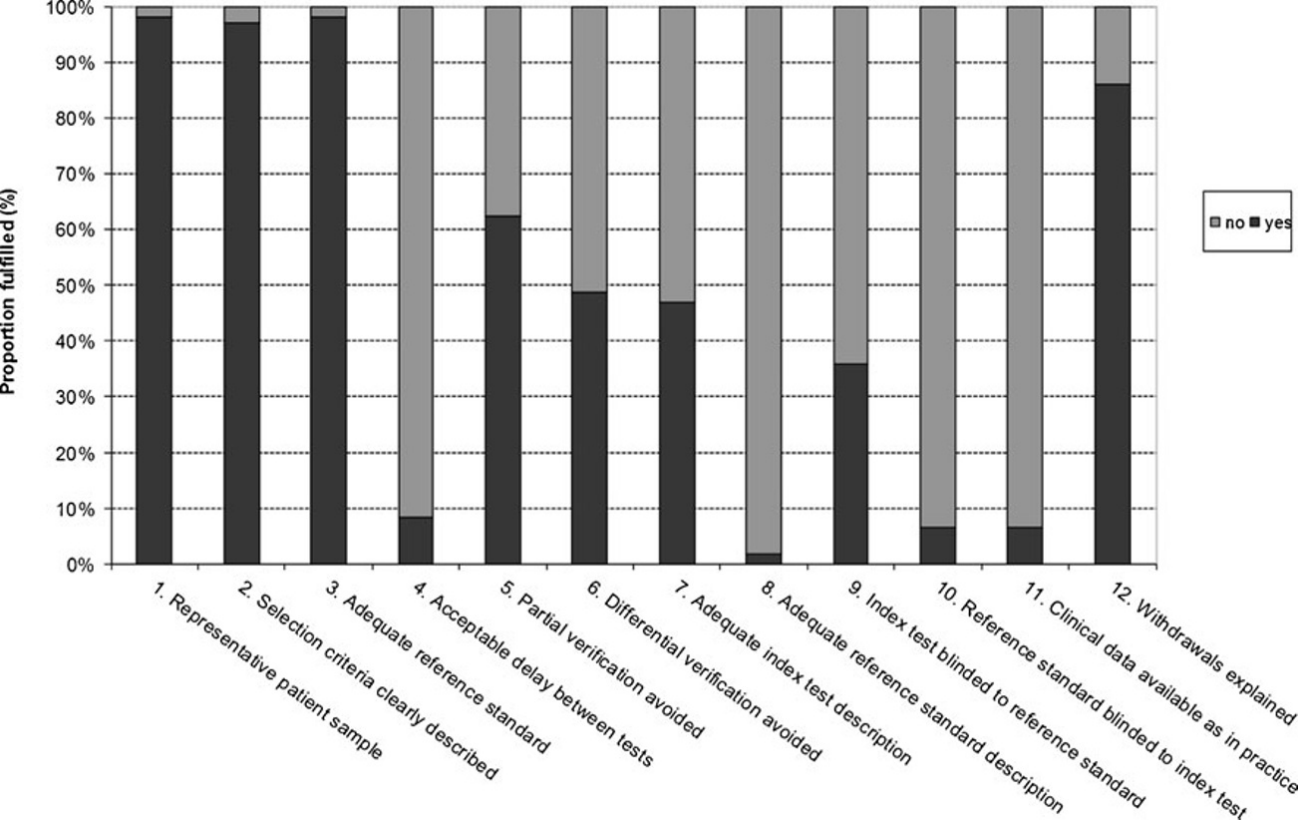
Overall quality assessment of all 109 included studies. Data are presented as *stacked bars* for each quality item of the modified Quality Assessment of the Studies of Diagnostic Accuracy Included in Systematic Reviews (QUADAS) tool

Quality assessment was performed by assessing quality scores for all studies, using 12 criteria adapted from the QUADAS tool [12]. Total quality scores in the included studies ranged from 25 to 75%. The average score of all studies was 50%, which is moderate, but indicates that these studies may have important limitations. In the quality assessment of these studies many negative scores were appointed because important details of study design were not reported (Fig. 2). In most studies, the patient sample was representative of the clinical setting in which DWI and ADC measurements are applied. The methods of patient enrolment were sufficiently described (in 97% of studies), and in many studies histopathological verification was available. However, in 38% of studies, histological diagnosis was not available for all lesions included in the analyses. Although in common practice it is not feasible to obtain histological verification of all (benign and malignant) lesions, and a reasonable reference standard is provided by follow-up of these lesions, partial verification bias may not be fully excluded. Reporting on methods of diffusion-weighted imaging and ADC measurement was not sufficient in a substantial fraction of the studies (53% of studies), although these data are important for the comparison of ADC measurements among studies. Furthermore, authors frequently failed to document whether blinding was used for the assessment of the index test (64% of studies) and reference test (94% of studies).

In the following section, a summary of the results will be presented per organ or body region. Due to the large number of studies reviewed, tables containing study characteristics could not be included in this paper but are provided in the Electronic Supplementary Material (ESM S1–S14).

### Intracranial

The articles regarding intracranial DWI addressed the following issues: differential diagnosis between cerebral abscesses and necrotic or cystic malignant tumours and between typical (benign) and atypical (malignant) meningiomas. Differentiation of high- and low-grade malignant brain tumours has also been studied extensively but lies beyond the scope of this review and will not be discussed.

**Fig. 3:**
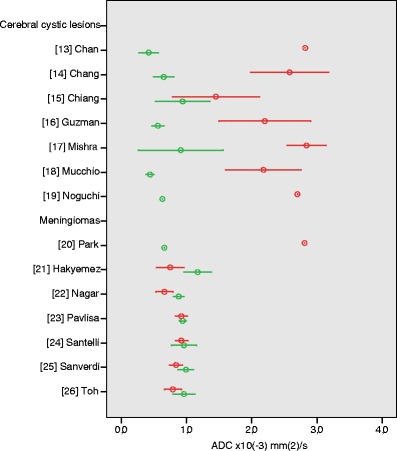
Apparent diffusion coefficient (ADC) values of intracranial lesions. Reported mean (*circles*) ADC ± 1 SD (*whiskers*) of cerebral cystic tumours (*red*) vs. cerebral abscesses (*green*) and malignant (*red*) vs. benign (*green*) meningiomas

We identified eight articles that compared mean ADC values of abscesses to malignant cerebral tumours with a cystic or necrotic component (ESM Table S1) [13–20]. Malignant lesions included in these studies were predominantly high-grade gliomas and metastases and a smaller number of low-grade gliomas and cerebral lymphomas. Quality scores of these articles were low to moderate, ranging from 25 to 50%. The studied populations ranged from 18 to 54 patients. Applied maximum *b*-values ranged from 972 to 1,200 s/mm^2^. All studies measured ADC values with placement of a region of interest (ROI) in the cystic component of abscesses and malignant tumours. These studies found that overall, cerebral abscesses show restricted diffusion with hyperintensity on DWI and low ADC values. Lowest reported mean ADC of abscesses was 0.42 ± 0.15 × 10^−3^ mm^2^/s and the highest reported mean ADC in these studies was 0.94 ± 0.42 × 10^−3^ mm^2^/s. For malignant lesions, reported mean ADC values ranged from 1.45 ± 0.67 to 2.96 × 10^−3^ mm^2^/s (Fig. 3). Three authors reported a significant difference between ADC values of cerebral abscesses and malignant tumours [15, 16, 19]; the other authors did not provide *P*-values.

Six articles discussed the contribution of DWI to the differentiation between histologic grades of meningiomas (ESM Table S2) [21–26]. Quality scores ranged from 42 to 67%. DWI was performed on a 1.0 T MR system in one study, 1.5 T in four studies, and 3.0 T in one study [26], and all authors applied a high maximum *b*-value of 800–1,000 s/mm^2^. All except one of the studies showed lower ADC values in atypical and malignant meningiomas than in typical meningiomas, with *P*-values <0.05 in three out of six studies [21, 22, 26]. However, considerable overlap existed between the types of meningiomas. Mean ADC values of typical meningiomas ranged from 0.88 ± 0.08 to 1.17 ± 0.21 × 10^−3^ mm^2^/s, mean ADC values of atypical and malignant meningiomas ranged from 0.66 ± 0.13 to 0.923 ± 0.085 × 10^−3^ mm^2^/s (Fig. 3).

### Salivary glands

**Fig. 4:**
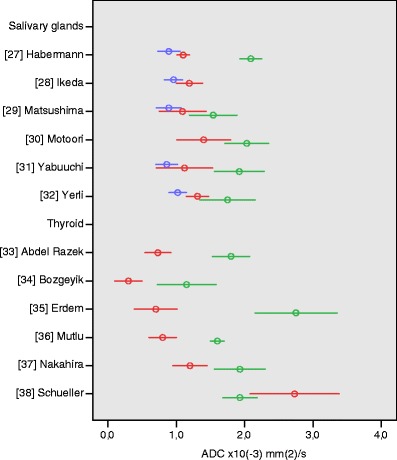
Apparent diffusion coefficient (ADC) values of salivary gland and thyroid lesions. Reported mean (*circles*) ADC ± 1 SD (*whiskers*) of malignant (*red*) vs. benign (*green*) thyroid tumours. Salivary gland malignant tumours (*red*) vs. pleomorphic adenomas (*green*) vs. Warthin tumours (*blue*)

We identified six articles that compared mean ADCs of pleomorphic adenomas, Warthin tumours, and salivary gland carcinomas (ESM Table S3) [27–32]. Two studies [29, 30] included lesions in all major salivary glands, four studies included only parotid gland lesions. The studies had moderate quality scores, ranging from 50 to 58%. All studies used a high maximum *b*-value of 1,000 s/mm^2^. Five authors [27, 29, 31–32] showed that pleomorphic adenomas have high mean ADC values, ranging from 1.54 ± 0.35 to 2.09 ± 0.16 × 10^−3^ mm^2^/s, compared to malignant tumours, which had mean ADC values ranging from 0.79 ± 0.33 to 1.40 ± 0.39 × 10^−3^ mm^2^/s (Fig. 4). This difference was significant in two out of five studies [25, 28]. The benign Warthin tumours were observed to have low mean ADC values, ranging between 0.89 ± 0.16 and 1.02 ± 0.13 × 10^−3^ mm^2^/s, comparable to the ADC values of malignant tumours.

### Thyroid

Six studies addressed the issue of differentiating benign and malignant thyroid nodules with DWI and ADC measurement (ESM Table S4) [33–38]. Quality scores of these studies ranged from 25 to 75%. Applied maximum *b*-values were 300 [34], 500 [33], 800 [38], and 1,000 [35–37]. All authors measured ADC values in solid (components of) lesions. The six included studies showed a marked variance in ADC values of thyroid nodules. In five studies [33–37], ADC values of thyroid carcinoma were significantly lower than ADC values of benign thyroid nodules. In these studies, mean ADC values of benign nodules ranged from 1.15 ± 0.43 to 2.75 ± 0.60 × 10^−3^ mm^2^/s, mean ADC values of malignant lesions ranged from 0.30 ± 0.20 to 1.20 ± 0.25 × 10^−3^ mm^2^/s (Fig. 4). However, one study [38] found a remarkably high mean ADC value in 16 thyroid carcinomas (2.73 ± 0.65 × 10^−3^ mm^2^/s), which was significantly higher than the mean ADC of benign thyroid adenomas (1.93 ± 0.25 × 10^−3^ mm^2^/s).

### Breast

**Fig. 5:**
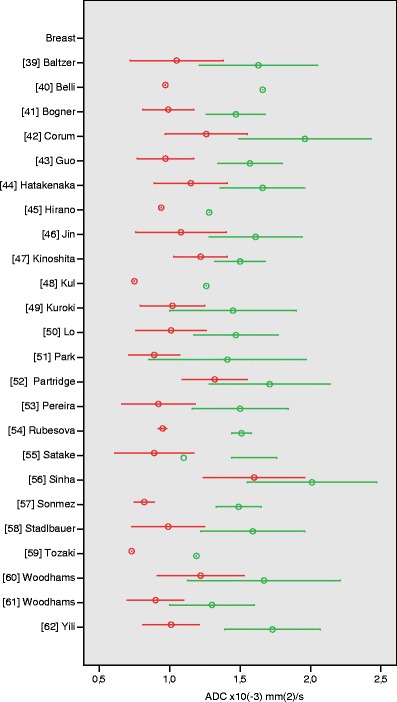
Apparent diffusion coefficient (ADC) values of breast lesions. Reported mean (*circles*) ADC ± SD (*whiskers*) of malignant (*red*) vs. benign (*green*) tumours

We identified 24 articles on the differentiation of breast lesions with ADC measurement (ESM Table S5) [39–62]. Quality scores ranged from 33 to 67%. The number of lesions included in the studies ranged from 22 to 262. Most studies applied high *b*-values of 1,000 s/mm^2^ (15 out of 24) or 1,500 s/mm^2^ (4 out of 24). Twenty studies showed statistically significant differences between ADC values of benign and malignant breast lesions. Among studies using maximum *b*-values of 700 or higher, mean ADC values of benign lesions ranged from 1.19 to 1.73 ± 0.34 × 10^−3^ mm^2^/s, whereas mean ADC values of malignant lesions ranged from 0.73 to 1.22 ± 0.31 × 10^−3^ mm^2^/s (Fig. 5). Higher mean ADC values were observed in three studies that applied lower *b*-values (maximum 290–600 s/mm^2^), for both benign (range 1.71 ± 0.43 to 2.01 ± 0.46 × 10^−3^ mm^2^/s) and malignant breast lesions (range 1.26 ± 0.29 to 1.60 ± 0.36 × 10^−3^ mm^2^/s) [42, 52, 56].

### Lung

**Fig. 6:**
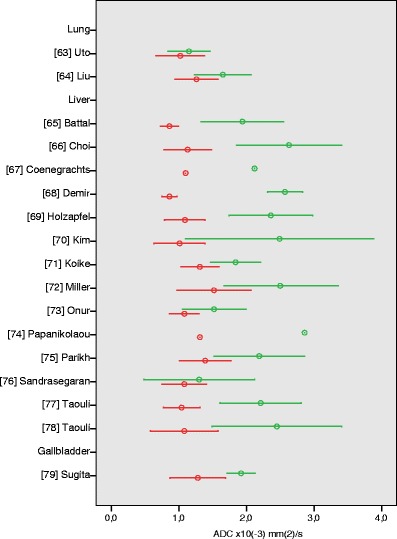
Apparent diffusion coefficient (ADC) values of lung, liver, gallbladder. Reported mean (*circles*) ADC ± SD (*whiskers*) of malignant (*red*) vs. benign (*green*) tumours

The systematic search revealed two studies on the ability of ADC values to discriminate between benign and malignant lung lesions (ESM Table S6). Uto et al. [63] studied 28 patients with pulmonary nodules, using *b*-values of 0 and 1,000 s/mm^2^. Quality score of this study was 67%. The calculated mean ADC for benign (inflammatory) nodules was 1.15 ± 0.31 × 10^−3^ mm^2^/s, and mean ADC for lung cancer was 1.02 ± 0.36 × 10^−3^ mm^2^/s (Fig. 6). There was no significant difference between benign pulmonary lesions and lung cancer (*P* = 0.388). A second study on ADC values in pulmonary nodules by Liu et al. [64], however, did show a significant difference between benign (inflammatory and noninflammatory) nodules and malignant nodules (*P* = 0.001). Mean ADC value of benign lesions was 1.65 ± 0.42, while malignant nodules had a mean ADC of 1.26 ± 0.32 × 10^−3^ mm^2^/s. The applied maximum *b*-value in this study was 500 s/mm^2^.

### Liver

Fourteen studies described ADC values of benign and malignant liver lesions (ESM Table S7) [65–78]. Quality scores ranged from 25 to 58%. All of these studies used single-shot EPI diffusion-weighted sequences. Seven studies applied maximum *b*-values of 400–600 s/mm^2^, while the other half of the studies applied maximum *b*-values of 800–1,000 s/mm^2^. Mean ADC values of benign hepatic lesions were higher than those of malignant lesions in all studies, of which 11 studies showed a statistically significant difference [65–70, 72–75, 78]. Mean ADC values of benign liver lesions ranged from 1.94 to 2.86 × 10^−3^ mm^2^/s, mean ADC values of malignant tumours ranged from 0.86 ± 0.11 to 1.52 ± 0.55 × 10^−3^ mm^2^/s (Fig. 6). In all but two of these studies, benign cysts were included in the group of benign lesions.

### Gallbladder

We identified only one article in which the differentiation of benign and malignant gallbladder lesions with ADC measurement is described (ESM Table S8). Sugita et al. [79] retrospectively studied ADC values of 14 benign and 15 malignant gallbladder lesions, with *b*-values of 0 and 1,000 s/mm^2^. Quality score of this study was 50%. Mean ADC of benign lesions was 1.92 ± 0.21 × 10^−3^ mm^2^/s, mean ADC of malignant lesions was 1.28 ± 0.41 × 10^−3^ mm^2^/s; the difference was statistically significant (*P* < 0.01) (Fig. 6).

### Pancreas

**Fig. 7:**
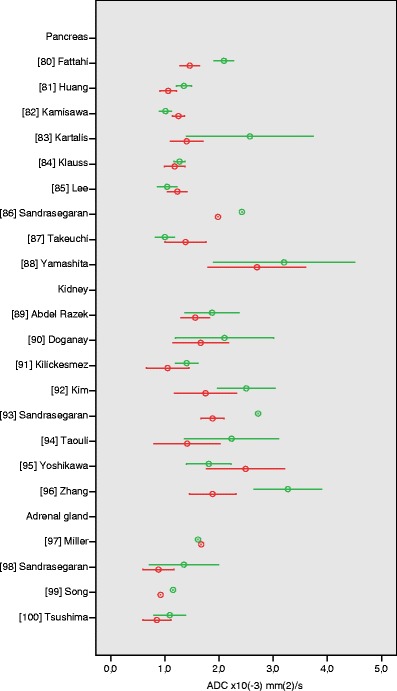
Apparent diffusion coefficient (ADC) values of pancreas, kidney, and adrenal gland lesions. Reported mean (*circles*) ADC ± SD (*whiskers*) of malignant (*red*) vs. benign (*green*) tumours

Several studies have been published on the differentiation of pancreatic cystic lesions with diffusion-weighted imaging and ADC measurement (ESM Table S9) [80–88]. Quality scores in these studies ranged from 33 to 58%. Five studies compared ADC values of benign mass-forming pancreatitis with ADC values of pancreatic cancer, and two studies did not specify the histological types of the investigated benign and malignant pancreatic lesions. These five studies on ADC measurement in pancreatic lesions show inconsistent results. Lee et al. [85] and Takeuchi et al. [87] found significantly lower mean ADC values in benign than in malignant pancreatic lesions, 1.04 ± 0.18 vs. 1.23 ± 0.18 × 10^−3^ mm^2^/s and 1.00 ± 0.18 vs. 1.38 ± 0.38 × 10^−3^ mm^2^/s, respectively (Fig. 7). Fattahi et al. [80] and Kartalis et al. [83] measured significantly higher mean ADC values in benign than in malignant lesions, 2.09 ± 0.18 and 2.57 ± 1.17 × 10^−3^ mm^2^/s versus 1.46 ± 0.18 and 1.40 ± 0.30 × 10^−3^ mm^2^/s, respectively. Yamashita et al. [88] also found mean ADC values of benign tumours (3.2 ± 1.3 × 10^−3^ mm^2^/s) to be higher than those of malignant tumours (2.7 ± 0.9 × 10^−3^ mm^2^/s), however, this was not statistically significant. ADC values measured by Yamashita et al. with relatively low *b*-values 0 and 300 s/mm^2^ were higher than the ADC values in the other studies.

### Kidney

ADC values of kidney tumours were described in six retrospective studies and two prospective studies (ESM Table S10) [89–96]. Quality scores of these studies ranged from 42 to 67%. Applied maximum *b*-values ranged from 400 to 1,000 s/mm^2^. All studies included benign renal cysts and seven out of eight studies showed significant differences between benign and malignant renal lesions. Lowest mean ADC values of benign lesions were reported for angiomyolipomas (1.40 to 1.81 × 10^−3^ mm^2^/s), and highest mean ADC values of benign lesions were reported for simple cysts (2.50 to 3.82 × 10^−3^ mm^2^/s). Mean ADC values of malignant renal lesions (mainly renal cell carcinomas) ranged from 1.05 to 2.49 × 10^−3^ mm^2^/s (Fig. 7).

### Adrenal gland

We found four studies that described ADC values of adrenal gland lesions (ESM Table S11). These studies had quality scores of 33–50% and applied high maximum *b*-values of 800–1,000 s/mm^2^. Sandrasegaran et al. [98] and Song et al. [99] evaluated benign pheochromocytomas and adenomas, which were reported to have significantly higher ADC values than malignant (metastatic) adrenal lesions (1.07 to 1.35 × 10^−3^ mm^2^/s versus 0.88 to 0.92 × 10^−3^ mm^2^/s, respectively). However, a larger study by Miller et al. [97] did not show a significant difference between benign and malignant adrenal gland lesions. Tsushima et al. [100] could only confirm a significant difference between adenomas and malignant pheochromocytomas, but not between adrenal adenomas and metastases (Fig. 7).

### Uterus

**Fig. 8:**
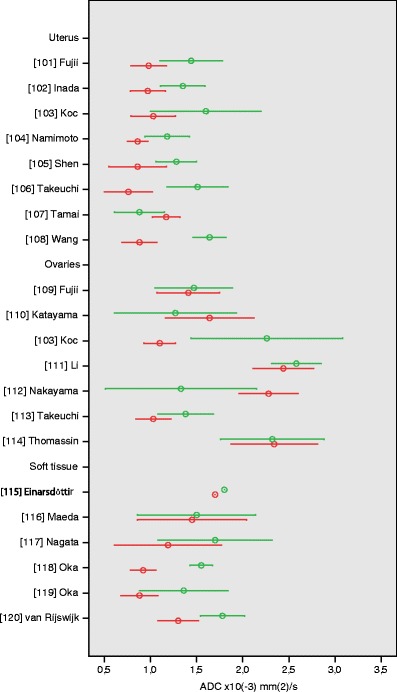
Apparent diffusion coefficient (ADC) values of uterus, ovary, and soft tissue lesions. Reported mean (*circles*) ADC ± SD (*whiskers*) of malignant (*red*) vs. benign (*green*) tumours

Eight studies have been published in which the differentiation between benign and malignant uterine tumours using ADC measurement was investigated (ESM Table S12) [101–108]. Quality scores of these studies ranged from 33 to 67%. All eight studies used high *b*-values (maximum 800–1,000 s/mm^2^) for diffusion-weighted imaging and showed comparable ADC values for uterine lesions. Benign lesions included in these studies were mainly leiomyomas, and the malignant lesions included were mainly endometrial carcinomas in five studies [101–103, 105, 108] and sarcomas in the other three studies [104, 106, 107]. Benign lesions had significantly higher mean ADC values (range 1.18 ± 0.24 to 1.64 ± 0.18 × 10^−3^ mm^2^/s) than malignant lesions (range 0.76 ± 0.26 to 1.17 ± 0.15 × 10^−3^ mm^2^/s) in seven out of eight studies (Fig. 8).

### Ovaries

Seven articles retrieved in our search studied ADC values in ovarian tumours (ESM Table S13) [103, 109–114]. Quality scores ranged from 50 to 67%. Applied maximum *b*-value was 1,000 s/mm^2^ in four studies, and 500–800 s/mm^2^ in three studies. In two studies, ADC values were measured in regions of interest placed in the cystic components of ovarian lesions [110, 112], two studies measured ADC values of solid components [109, 113] and three studies measured ADC values of both cystic and solid components [103, 111, 114]. Mean ADC values of cystic components of benign ovarian lesions ranged from 1.24 ± 0.46 to 2.32 ± 0.56 × 10^−3^ mm^2^/s, mean ADC values of cystic components of malignant lesions ranged from 1.64 ± 0.48 to 2.34 ± 0.47 × 10^−3^ mm^2^/s (Fig. 8). Only one study [112] found a significant difference between mean ADC values of cystic components of benign (1.33 ± 0.82 × 10^−3^ mm^2^/s) and malignant tumours (2.28 ± 0.32 × 10^−3^ mm^2^/s). The ADC values of solid components of benign ovarian lesions (range 1.15 ± 0.55 to 1.47 ± 0.42 × 10^−3^ mm^2^/s) and malignant ovarian lesions (range 1.14 ± 0.28 to 1.41 ± 0.34 × 10^−3^ mm^2^/s) were not significantly different [109, 114].

### Soft-tissue

Six articles have been published in which the application of diffusion-weighted imaging and ADC measurement to the characterisation of soft-tissue tumours is discussed (ESM Table S14) [115–120]. Quality scores of these articles ranged from 33 to 67%. One article focused on the differentiation between chronic expanding hematomas (CEH) and malignant soft-tissue tumours and observed significantly (*P* < 0.01) higher mean ADC values in CEH (1.55 ± 0.121 × 10^−3^ mm^2^/s) than in malignant tumours (0.92 ± 0.139 × 10^−3^ mm^2^/s) [118]. Five authors studied ADC values in various benign and malignant tumours [115–117, 119, 120]. Mean ADC values of benign soft-tissue tumours (range of mean ADC values 1.36 ± 0.48 to 1.80 × 10^−3^ mm^2^/s) overlapped with those of malignant soft-tissue tumours (range of mean ADC values 0.88 ± 0.20 to 1.70 × 10^−3^ mm^2^/s). Only one study found a significant difference between benign desmoid tumours and malignant soft-tissue tumours (Fig. 8) [118].

## Discussion

Since its development in the early 1990s, the application of diffusion-weighted imaging has expanded from intracranial to extracranial disease and from detection of brain ischemia to assessment of tumour masses. Its potential additional value in oncological imaging lies in the fact that it provides functional tissue information, which can be combined with anatomical MR images to improve the specificity of lesion characterisation. Besides the qualitative assessment of signal intensity in DWI, images can be assessed quantitatively by the measurement of ADC values. We performed a systematic review of the recent literature in order to obtain an insight into the value of DWI and ADC measurement in differentiating benign from malignant tumour masses.

### Additional value of ADC measurement in tumour characterisation

In DWI, tissue contrast is obtained through differences in free water motion between various tissue types and between normal and pathological tissues. The functional information provided by DWI and ADC measurement may be of value in tumour characterisation, complementary to the anatomical information obtained with conventional MRI sequences. Because of its high contrast-to-noise ratio, lesions with restricted diffusion are usually easily recognised on diffusion-weighted images [10]. A drawback of DWI is the relatively low spatial resolution of the images, compared with conventional T1- or T2-weighted MR images. Small lesions (i.e., below spatial resolution) may not be visible on DWI and ADC maps [51], and partial volume averaging is more likely to occur. Several of the reviewed studies excluded small lesions for this reason. Similarly, lesions with a degree of diffusivity equal to the surrounding normal tissue may not be easily distinguished on DWI images and ADC maps. Thus, not all lesions are suitable for ADC measurement.

The lowest mean ADC value of extracerebral benign lesions (0.86 × 10^−3^ mm^2^/s) was reported for Warthin tumours of the parotid glands, while benign renal cysts showed the highest mean ADC value (3.82 × 10^−3^ mm^2^/s). The lowest and highest mean ADC values reported for malignant lesions were 0.30 × 10^−3^ mm^2^/s in thyroid carcinomas and 2.70 × 10^−3^ mm^2^/s in malignant pancreatic lesions, respectively. The observed wide variation in ADC values within benign and malignant tumours can be partly explained by the wide variety of histological subtypes of proliferative tumours. In most malignant tumours, diffusion is restricted due to increased cellular density and decreased extracellular matrix volume, which impede free motion of water molecules [10, 121, 122]. However, some malignant tumours show increased diffusion due to an increase in intratumoural water content, which is the case in intratumoural edema and in cystic tumour components. The degree of serous or mucinous content and intratumoural hemorrhage also influences the signal intensity and ADC value through their effect on restriction of free proton diffusion and magnetic susceptibility of the tumour tissue. Furthermore, loss of cell membrane integrity in necrotic tumours may result in increased diffusion [10, 121, 122]. This was also demonstrated in the studies that compared ADC values of cerebral necrotic/cystic tumours and cerebral abscesses, in which high mean ADC values of necrotic tumours (ranging from 2.58 ± 0.60 to 2.84 ± 0.30 × 10^−3^ mm^2^/s) were reported [13–20]. In cerebral abscesses restricted diffusion was observed, with mean ADC values ranging from 0.42 ± 0.15 to 0.91 ± 0.65 × 10^−3^ mm^2^/s [13–20]. It is postulated that the restricted diffusion in abscesses is attributable to high viscosity of pus resulting from high protein and different types of viable or dead cells, along with necrotic tissue and bacteria [123]. Mean ADC values in other benign or malignant cystic lesions in the body were comparable to the high values in malignant cerebral cystic tumours (1.45 to 2.96 × 10^−3^ mm^2^/s) [13–20]. For example, the following ranges of mean ADC values were described in various cystic lesions: 2.35 ± 0.08 to 2.65 ± 0.30 × 10^−3^ mm^2^/s in benign cystic breast lesions [43, 56], 1.902 to 3.63 × 10^−3^ mm^2^/s in simple liver cysts [66, 68–71, 75, 76, 78], 1.33 ± 0.82 to 2.32 ± 0.56 × 10^−3^ mm^2^/s in cystic components of benign ovarian tumours [114] and 2.34 ± 0.47 × 10^−3^ mm^2^/s in cystic components of malignant ovarian tumours [114].

We observed significant differences in reported ADC values between benign and malignant tumours in the following tissues: brain abscesses vs. cystic brain tumours, meningiomas, breast, liver, and uterus. However, many studies showed considerable overlap between ADC values of benign and malignant tumours. The presence of overlap complicates potential prospective usage of these quantitative measurements, which calls for the use of “artificial” cut-off values.

In some organs, such as the salivary glands, thyroid, lungs, pancreas, and soft tissue, the reported data on the value of ADC measurements showed contradictory results. ADC measurement in these organs is unlikely to contribute to the differentiation between benign and malignant lesions. In the pancreas, ADC measurements fail to differentiate cysts. Furthermore, it is generally accepted that we need both high *b*-value DWI and ADC mapping for the diagnosis of solid pancreatic lesions. Likewise, we have observed widely varying reported ADC values of benign and malignant ovarian lesions. In cystic ovarian lesions, conventional MR imaging is often not conclusive and differentiation by ADC measurement would be useful. However, the reviewed studies that compare ADC values in cystic components of benign and malignant ovarian tumours showed contradictory results. Moreover, ADC values of solid components of benign and malignant cystic ovarian lesions were not significantly different [103, 109–114]. In several breast and liver studies, simple benign cysts were enrolled, which are well recognised on conventional (T1- and T2-weighted) imaging and usually do not cause diagnostic dilemmas. Including these cysts may overestimate the ability of ADC measurements to discriminate benign from malignant lesions. Another limitation of many studies included in this review was that benign lesions in particular were frequently confirmed by other imaging modalities and follow-up, without histological assessment. This is a common procedure in daily practice but may cause bias in the comparison of ADC values of benign and malignant tumours.

### Technical aspects of DWI and ADC measurement

Quality of diffusion-weighted images and ADC measurements may vary when different MR imaging parameters and MR systems are used. The most important limitations of DWI are the low SNR and susceptibility to artefacts [10]. Strategies to optimise image quality often incorporate the use of parallel imaging techniques, fat-suppression techniques, and signal averaging. Additional factors that can influence measured ADC values are use of breath-hold, respiratory triggering, or free-breathing acquisition and direction of diffusion gradients. Diffusion-weighting gradients are commonly applied in three orthogonal directions, which is desirable particularly in tissues with anisotropic orientation such as brain and kidney, in which ADC values may differ among the x, y, and z directions [10].

Another important factor in DWI is the maximum *b*-value. When low *b*-values are applied, the ADC values tend to be higher due to the contribution of perfusion. This was shown by several studies that applied low *b*-values to ADC measurement of breast lesions [42, 52, 56]. On the other hand, among the reviewed studies on ADC values of liver lesions, the ADC values did not clearly differ between high and low *b*-values. In malignant tumours, a higher percentage of microvessels is present than in benign tissue [124]. Accordingly, perfusion may artificially increase the ADC in malignant lesions and complicate differentiation. Therefore, if ADC measurement is performed to differentiate tissues by their water diffusion characteristics exclusively, applying high maximum *b*-values may be preferable. However, signal-to-noise ratios decrease as the *b*-value increases, thus limiting the maximum *b*-value. Another way of minimising contribution of perfusion to the ADC value is to select minimum *b*-values higher than 0 s/mm^2^ (e.g., 100 s/mm^2^), which was done in several reviewed studies [19, 41, 69]. Optimal *b*-values should be chosen for each organ, however, no consensus or guidelines are available for that purpose. In the reviewed articles, various methods of ADC measurement have been used. ADC measurement is performed by placing regions of interest (ROI) in the lesion on the acquired ADC maps. Variations occurred in applied size and shape of the ROI, use of T1/T2 MR images for guidance of ROI placement on ADC map, and averaging of multiple ROIs. As the ROI placement is usually performed manually, training is required to optimise the reproducibility (minimise interobserver variation) of ADC measurement. Furthermore, in case of lesions with both solid and cystic components, a consensus on localisation of ROI (in solid or cystic part of lesion) should be established.

### Study limitations

Selection of eligible articles and assessment of study quality was performed by one author only, which could be considered a limitation. However, in a study by Whiting et al., reproducibility of the QUADAS instrument has been reported to be good [125]. Three reviewers independently rated the quality of 30 studies using QUADAS. The proportion of agreements between each reviewer and the final consensus rating was assessed. This was done for all QUADAS items combined and for each individual item. Over all items, the agreements between each of the reviewers and the final consensus rating were 91, 90, and 85%. The results for individual QUADAS items ranged from 50 to 100% with a median value of 90% [125].

As this study aimed to evaluate the potential of ADC measurements to differentiate between benign and malignant lesions in the body, we included a large number of studies on ADC measurement in a variety of organs. Consequently, heterogeneity in study methods and applied MRI parameters was observed, which precluded the performance of statistical meta-analysis.

### Towards standardisation of ADC measurement and reporting

If ADC measurements are to be routinely used in clinical practice, standardisation of protocols across institutions is required in order to improve reproducibility. Differences in MR protocols and in histological types of lesions enrolled in the studies contribute to the variation in reported ADC values. In order to obtain reliable data on the value of ADC measurement in clinical practice, two issues need to be addressed. Firstly, applied MR parameters differ among hospitals, which complicates a direct comparison of ADC values. Therefore, standardised diffusion-weighted MRI protocols need to be established to ensure reproducibility at different centers [126, 127]. In particular, the use of *b*-values and method of ADC measurement should be standardised for clinical application, adjusted to each organ of interest. Secondly, due to incomplete reporting of study methods, comparison of ADC values among studies becomes challenging. Detailed reporting of patient population and data collection is important to assess generalisability of study results. Notably, standardised acquisition and reporting may not fully solve this problem, as differences in SNR and image quality across various MR systems may still cause some variation in measured ADC values. Padhani et al. have provided useful recommendations for the use of DWI as a cancer biomarker, both for the choice of methods of measurement and analysis and for the reporting of data [127]. Furthermore, the Standards for Reporting of Diagnostic Accuracy (STARD) criteria provide a good guideline for the reporting of study methods in diagnostic accuracy studies [128]. When key DWI parameters are well reported, differences in reported ADC values may be easier to evaluate. In addition, we suggest a short checklist of DWI parameters that can serve as a guideline for reporting in studies on ADC measurement for tumour characterisation (Table 3).

**Table 3:** Checklist for reporting diffusion-weighted imaging (DWI) technique in studies on apparent diffusion coefficient (ADC) measurement in tumour characterisation (recommended minimum requirements)

DWI parameters
Field strength (T)
Coil type (i.e., built-in body coil/surface coils)
Pulse sequence [e.g., single-shot spin-echo/single-shot double spin-echo/multi-shot spin-echo, echo planar imaging (EPI)/non-EPI, etc.]
Repetition time, echo time (ms)
*b*-values (s/mm^2^)
Directions of diffusion-weighting gradients
Fat saturation technique (e.g., fat saturation, inversion recovery, water selection only, etc.)
Number of excitations
Parallel acquisition factor
Echo train length
Respiratory motion correction technique (i.e., breath-hold/respiratory gating/none)
Cardiac motion correction technique (i.e., ECG triggering/finger pulse triggering/none)
Voxel size (mm^3^)
Receiver bandwidth
Acquisition of DWI data before or after intravenous contrast administration
Method of ADC calculation
Applied model for ADC calculation (e.g., monoexponential, biexponential, etc.)
*b*-values that were used to calculate the ADC
Method of ADC measurement
Description of which portion of the tumour was measured (e.g., whole tumour, only enhancing and/or solid portions, etc.)
Description of ROI margins (i.e., distance from tumour periphery)
ROI shape and size (fixed or variable)
Single or multiple slice ROI measurement
Verification of ROI position on diffusion-weighted images that were used to calculate the ADC map

*T* Tesla, *EPI* echo planar imaging, *ECG* electrocardiogram, *ROI* region of interest

## Conclusion

Reported ADC values among studies and between benign and malignant lesions differ considerably. In several tumours, such as brain abscesses vs. cystic brain tumours, meningiomas, and breast, liver, and uterine tumours, ADC measurement may be of value to discriminate benignancy from malignancy. However, in other organs, such as the salivary glands, thyroid, lungs, pancreas, and soft tissue, the ADC value does not appear to contribute to tumour characterisation.

One of the challenges that must be faced to enable widespread adoption of ADC measurement in clinical practice is standardisation of study methods and reporting. The development of organ-specific guidelines for DWI acquisition and ADC measurement and checklists for reporting of results may facilitate comparison of study results and contribute to the implementation of ADC measurement for tumour characterisation in the clinical setting.

## Electronic Supplementary Materials

Below is the link to the electronic supplementary material.

ESM 1 (DOC 238 kb)
