# Supplementary material for: Apparent diffusion coefficient measurements in the differentiation between benign and malignant lesions: a systematic review
Source: Insights Imaging. 2012 Jun 7;3(4):395–409. doi: 10.1007/s13244-012-0175-y (PMC3481080; doi:10.1007/s13244-012-0175-y)
Supplement: Supplementary file 1 — (DOC 238 kb) [file 13244_2012_175_MOESM1_ESM.doc]

**Electronic Supplementary Material**

**Apparent diffusion coefficient measurements in the differentiation between benign and malignant lesions: a systematic review.**

**Tables S1-S14: Study Characteristics Tables**

Table S1: Cerebral abscess vs. tumour

| Study | N benign | N malignant | b-values  s/mm2 | ADC benign x10-3 mm­­­2/s | ADC malignant x10-3 mm­­­2/s | p-value | Tumour types | Modified QUADAS items with positive score | Quality score % |
| --- | --- | --- | --- | --- | --- | --- | --- | --- | --- |
| [13] Chan  2002 | 7 | 18 | 200, 600, 1000 | 0.42±0.15 (central region) | Metastasis 2.96, lymphoma 2.3-2.5, glioblastoma multiforme 2.82 | ? | Benign: 7 pyogenic abscess  Malignant: 8 cystic metastasis, 6 glioblastoma multiforme, 2 cerebral lymphoma, 2 CNS lymphoma with AIDS | 1, 2, 3, 5, 6 | 42 |
| [14] Chang  2002 | 14 | 19 | 0, 1000 | 0.65±0.16  (central region) | 2.58±0.60 | ? | Benign: 11 pyogenic abscess Malignant: 14 metastasis, 3 glioblastoma multiforme, 1 anaplastic astrocytoma, 1 fibrillary astrocytoma | 1, 3, 6, 9 | 33 |
| [15] Chiang  2009 | 20 | 26 | 1000 | 0.94±0.42 (central region) | 1.45±0.67 | 0.004 | Benign: 20 pyogenic brain abscess  Malignant: 14 glioblastoma multiforme, 12 metastasis | 1, 2, 3 | 25 |
| [16] Guzman  2002 | 11 | 16 | 50, 972 | 0.56±0.09  (central part) | low-grade gliomas: 1.51±0.2, high-grade gliomas: 2.2±0.7, metastases: 1.2±0.2 | absces vs low-grade glioma or meta: p<0.05, abces vs high-grade glioma: p<0.00 | Benign: 11 abscess  Malignant: 7 glioblastoma, 4 metastasis, 3 low-grade glioma, 1 oligoastrocytoma, 1 anaplastic astrocytoma | 1, 2, 3, 5, 6, 12 | 50 |
| [17] Mishra  2004 | 29 | 23 | 0, 1000 | 0.91±0.65  (cystic cavity) | 2.84±0.30 | ? | Benign: 26 pyogenic abscesses, 3 tubercular abscesses Malignant: 7 pilocytic astrocytoma, 5 hemangioblastoma, 3 glioblastoma, 3 anaplastic astrocytoma, 1 ganglioglioma, 1 metastatic adenocarcinoma, 3 benign cyst (neurocysticercosis) | 1, 2, 3, 5, 6, 12 | 50 |
| [18] Mucchio  2008 | 5 | 15 | 0, 1000 | 0.44±0.06  (lesion core) | glioblastoma: 2.18±0.58, metastase: 2.37±0.50 | ? | Benign: 5 pyogenic abscess  Malignant:10 glioblastoma, 5 metastasis | 1, 3, 4 | 25 |
| [19] Noguchi  1999 | 6 | 18 | 30, 1200 | 0.63  (cystic part) | 2.70 | sigificant difference between ADC of brain abscesses and solid/cystic/necrotic parts of malignant tumours. | Benign: 6 abscess  Malignant: 12 metastasis, 6 high-grade glioma | 1, 3, 6 | 25 |
| [20] Park  2000 | 6 | 12 | 0, 1000 | 0.66  (cystic portion) | malignant glioma: 2.81, metastasis: 2.20 | ? | Benign: 6 abscess  Malignant: 10 metastasis, 2 malignant glioma | 1, 2, 3 | 25 |

Note: QUADAS: Quality Assessment of the Studies of Diagnostic Accuracy Included in Systematic Reviews, CNS: central nervous system, AIDS: acquired immunodeficiency syndrome

Table S2: Benign vs. malignant meningioma

| Study | N benign | N malignant | b-values  s/mm2 | ADC benign x10-3 mm­­­2/s | ADC malignant x10-3 mm­­­2/s | p-value | Tumour types | Modified QUADAS items with positive score | Quality score % |
| --- | --- | --- | --- | --- | --- | --- | --- | --- | --- |
| [21] Hakyemez  2006 | 32 | 7 | 0, 1000 | 1.17±0.21 | 0.75±0.21 (atypical+ malignant) | <0.001 | Benign: 32 typical meningioma  Malignant:6 atypical meningioma, 1 malignant meningioma | 1, 2, 3, 5, 6, 11, 12 | 58 |
| [22] Nagar  2008 | 23 | 25 | 0, 1000 | 0.88±0.08 | 0.66±0.13 | <0.0001 | Benign: 23 benign meningioma  Malignant: 25 atypical/malignant meningioma | 1, 2, 3, 4, 5, 10, 12 | 58 |
| [23] Pavlisa  2008 | 21 | 5 | 0, 500, 1000 | 0.939±0.059 | 0.917±0.087 | >0.05 | Benign: 21 typical meningioma  Malignant: 5 atypical meningioma | 1, 2, 3, 5, 6, 10, 12 | 58 |
| [24] Santelli  2010 | 79 | 23 | 0, 800 | 0.964±0.192 | 0.923±0.085 | 0.3 | Benign: 79 benign meningioma  Malignant: 22 atypical meningioma, 1 malignant meningioma | 1, 2, 3, 5, 7, 8, 9, 12 | 67 |
| [25] Sanverdi  2011 | 135 | 42 | 3 b-values max. 1000 | 0.99±0.12 | 0.84±0.1 | Not significant | Benign: 135 benign meningioma  Malignant: 37 atypical meningioma, 5 malignant meningioma | 1, 2, 3, 5, 7, 9, 12 | 58 |
| [26] Toh  2008 | 12 | 12 | 0, 1000 | 0.964±0.172 | 0.791±0.129 | 0.011 | Benign: 12 classical meningioma  Malignant: 12 atypical meningioma | 1, 2, 3, 5, 6 | 42 |

Note: QUADAS: Quality Assessment of the Studies of Diagnostic Accuracy Included in Systematic Reviews

Table S3: Salivary glands

| Study | N benign | N malignant | b-values  s/mm2 | ADC benign x10-3 mm­­­2/s | ADC malignant x10-3 mm­­­2/s | p-value | Tumour types | Modified QUADAS items with positive score | Quality score % |
| --- | --- | --- | --- | --- | --- | --- | --- | --- | --- |
| [27] Habermann 2009 | 87 | 49 | 0, 500, 1000 | PA: 2.09±0.16, WT: 0.89±0.16 | Mucoepidermoid carcinoma: 1.05±0.03, salivary duct carcinoma: 1.1. ±0.09, acinic cell carcinoma: 0.79±0.33, basal cell adenocarcinoma: 0.96±0.29 | PA significantly higher than all other entities, WT only significantly different from salivary duct ca. | Benign: 43 PA, 32 WT, 6 myoepithelial adenoma, 3 lipoma, 1 basal cell adenoma, 1 cystadenoma, 1 inverted ductal papilloma  Malignant: 16 mucoepidermoid carcinoma, 11 SDC, 10 acinic cell carcinoma, 9 basal cel adenocarcinoma, 1 adenoid cystic carcinoma, 1 epithelial-myoepithelial carcinoma, 1 CEPA | 1, 2, 3, 4, 5, 6, 12 | 58 |
| [28] Ikeda 2004 | 19 | 17 | 0, 1000 | WT: 0.96±0.13 | 1.19±0.19 | <0.01 | Benign: 19 WT Malignant: 4 acinic cell adenocarcinoma, 5 SDC, 23 CEPA, 2 adenoid cystic carcinoma, 1 SCC, 1 basal cell carcinoma | 1, 2, 3, 5, 6, 12 | 50 |
| [29] Matsushima 2007 | 17 | 15 | 5, 1000 | 1.40±0.43 (PA: 1.54±0.35, WT: 0.89±0.18) | 1.09±0.34 | >0.05 (benign vs. Malignant), >0.05 (WT vs malignant) | Benign: 12 PA, 4 WT, 1 neurofibroma  Malignant: 4 adenoid cystic carcinoma, 2 malignant lymphoma, 2 CEPA, 1 myoepithelial carcinoma, 1 lymphoepithelial carcinoma, 1 SCC, 1 undifferentiated carcinoma, 1 SDC, 1 malignant melanoma, 1 myxoid liposarcoma | 1, 2, 3, 5, 6, 12 | 50 |
| [30] Motoori 2004 | 22 | 13 | 0, 1000 | PA: 2.03±0.32 | 1.40±0.39 | <0.01 | Benign: 22 pleomorphic adenoma  Malignant: 13 malignant parotid gland tumours | 1, 2, 3, 5, 6, 12 | 50 |
| [31] Yabuuchi 2008 | 36 | 14 | 0, 500, 1000 | PA: 1.92±0.36, WT: 0.86±0.16 | carcinomas 1.12±0.41, malignant lymphomas 0.88±0.07 | ? | Benign: 20 PA, 14 WT  Malignant: 3 CEPA, 3 acinic cell carcinoma, 2 non-Hodgkin lymphoma, 2 adenoid cystic carcinoma, 1 adenocarcinoma, 1mucoepidermoid cyste, SDC, 1 SCC | 1, 2, 3, 5, 6, 9, 12 | 58 |
| [32] Yerli  2010 | 16 | 5 | 0, 500, 1000 | Adenoma: 1.75±0.40  WT: 1.02±0.13 | 1.31±0.16 | Benign vs. malignant not significant | Benign: 8 WT, 8 PA, 2 basal cel adenoma  Malignant: 3 adenoid cystic carcinoma, 2 adenocarcinoma | 1, 2, 3, 5, 7, 12 | 50 |

Note: QUADAS: Quality Assessment of the Studies of Diagnostic Accuracy Included in Systematic Reviews, PA: pleomorphic adenoma, WT: Warthin tumour, SCC: squamous cell carcinoma, SDC: salivary duct carcinoma, CEPA: carcinoma ex pleomorphic adenoma

Table S4: Thyroid

| Study | N benign | N malignant | b-values  s/mm2 | ADC benign x10-3 mm­­­2/s | ADC malignant x10-3 mm­­­2/s | P-value | Tumour types | Modified QUADAS items with positive score | Quality score % |
| --- | --- | --- | --- | --- | --- | --- | --- | --- | --- |
| [33] Abdel Razek  2008 | 56 | 7 | 0, 250, 500 | 1.8±0.27 (cysts: 1.9±0.38) | 0.73±0.19 | 0.001 | Benign: 47 adenomatous nodule, 6 follicular adenoma, 8 thyroid cyst  Malignant: 4 papillary carcinoma, 3 follicular carcinoma | 1, 2, 3, 4, 5, 6, 10, 11, 12 | 75 |
| [34] Bozgeyik  2009 | 88 | 5 | 100, 200, 300 | 1.15±0.43 (solid parts) | 0.30±0.20 | <0.05 | Benign: NA  Malignant: NA | 1, 2, 4, 6, 11, 12 | 50 |
| [35] Erdem  2010 | 52 | 16 | 0, 1000 | 2.7453±0.6011 (solid parts) | 0.6952±0.3125 | 0.0001 | Benign: NA (no cysts)  Malignant: 6 papillary carcinoma, 1 follicular neoplasia, 1 anaplastic carcinoma, 1 lymphoma | 1, 2, 3 | 25 |
| [36] Mutlu  2012 | 46 | 5 | 0, 50, 400, 1000 | 1.6±0.1 | 0.8±0.2 | significant | Benign: NA  Malignant: NA | 1, 2, 3, 4, 6, 7 | 50 |
| [37] Nakahira  2011 | 23 | 19 | 0, 1000 | 1.93±0.37 | 1.20±0.25 | <0.01 | Benign: 16 adenomatous goiter, 7 follicular adenoma  Malignant: 12 papillary carcinoma, 4 follicular carcinoma, 2 medullary carcinoma, 2 metastatic breast cancer | 1, 2, 3, 5, 7, 9, 12 | 58 |
| [38] Schueller-Weidekam  2010 | 15 | 20 | 0, 800 | Adenoma: 1.93±0.25, Thyroiditis: 3.46±0.40 | 2.73±0.65 | Adenoma vs carcinoma p<0.05, thyroiditis vs carcinoma p>0.05 | Benign: 11 adenoma, 4 Hashimoto thyroiditis  Malignant: 5 medullary carcinoma, 10 papillary carcinoma, 4 follicular carcinoma, 1 mixed medullary/papillary carcinoma | 1, 2, 3, 5, 6, 8, 12 | 58 |

Note: QUADAS: Quality Assessment of the Studies of Diagnostic Accuracy Included in Systematic Reviews, NA: not available

Table S5: Breast

| Study | N benign | N malignant | b-values  s/mm2 | ADC benign x10-3 mm­­­2/s | ADC malignant x10-3 mm­­­2/s | P-value | Tumour types | Modified QUADAS items with positive score | Quality score % |
| --- | --- | --- | --- | --- | --- | --- | --- | --- | --- |
| [39] Baltzer 2009 | 35 | 39 | 0, 750, 1000 | EPI: 1.625±0.419 HASTE: 1.670±0.396 | EPI: 1.053±0.325 HASTE: 1.092±0.378 | <0.001 | Benign: 13 FA, 3 PP, 11 FCC, 1 BPh, 3 galactophoritis, 4 other benign changes  Malignant: 30 IDC, 4 mixed IDC/lLC, 5 DCIS | 2, 3, 6, 10, 11, 12 | 50 |
| [40] Belli  2010 | 26 | 100 | 0, 1000 | 1.66 | 0.97 | <0.0001 | Benign: 9 FA, 3 complicated cysts, 4 simple cyst, 1 BPh, 1 benign radial scar, 3 atypical ductal hyperplasia, 1 tubular adenoma, 1 PP, 1 gynaecomastia, 1 giant angioma, 1 abscess  Malignant: 13 carcinoma in situ, 12 ILC, 68 IDC, 4 poorly differentiated carcinoma, 1 MPh, 1 papillary carcinoma, 1 MuC | 1, 2, 3, 5, 6, 9, 12 | 58 |
| [41] Bogner  2009 | 17 benign  20 cystic | 24 | 50, 850 | Solid benign: 1.47±0.21,  cystic: 2.64±0.30 | 0.99±0.18 | <0.001 | Benign: 5 FA, 3 FCC, 1 adenosis, 8 not specified  Malignant: 17 IDC, 4 ILC, 2 DCIS, 1 MPh | 1, 2, 3, 6, 9, 12 | 50 |
| [42] Corum 2009 | 10 | 15 | 7 b-values from 7.16-458 | 1.96±0.47 | 1.26±0.29 | <0.001 | Benign: 3 FA, 3 FCC, 1 fibrosis  Malignant: 14 IDC, 1 ILC | 1, 2, 3, 5, 6, 12 | 50 |
| [43] Guo  2002 | 24 | 31 | 0, 250, 500, 750, 1000 | benign solid lesions: 1.57±0.23, cysts 2.35±0.08 | 0.97±0.20 | solid benign vs malignant p=0.000 | Benign: 18 FA, 4 cysts, 1 PP, 1 ductal ectasia  Malignant: 25 IDC, 2 DCIS, 3 MeC, 1 scirrhous adenocarcinoma | 1, 2, 3, 12 | 33 |
| [44] Hatakenaka 2008 | 16 | 117 | 0, 500, 1000 | FA:1.66±0.30 | IDC 1.15±0.26 | <0.001 | Benign: 16 FA Malignant: 117 IDC, 3 medullary carcinoma, 4 MuC | 1, 2, 3, 5, 6, 7, 12 | 58 |
| [45] Hirano  2012 | 27 | 48 | 50, 800, 1500 | 1.28 | 0.94 | <0.01 | Benign: 3 FCC, 12 FA, 10 PP, 2 phyllodes tumor  Malignant: 2 DCIS, 41 IDC, 4 MuC, 1 ILC | 1, 2, 3, 4, 5, 7, 9, 12 | 67 |
| [46] Jin  2010 | 20 | 40 | 0, 600, 1000 | b-1000:  1.61±0.33 | b-1000:  1.08±0.32 | <0.05 | Benign: 10 fibroadenosis, 4 FA, 2 PP, 1 ductal ectasia  Malignant: 33 IDC, 2 DCIS, 2 MuC, 1 ILC, 1 malignant lymphoma | 1, 2, 3, 4, 7, 9, 12 | 58 |
| [47] Kinoshita 2002 | 6 | 16 | 0, 700 | 1.495±0.181 | IDC 1.216±0.189 | <0.01 | Benign: 6 FA Malignant: 16 IDC | 1, 2, 3, 12 | 33 |
| [48] Kul  2011 | 37 | 47 | 50, 1000 | (median)  1.26 | (median)  0.75 | <0.001 | Benign: 16 FA, 10 FCC, 6 benign epithelial hyperplasia, 2 mastitis, 2 PP, 1 fat necrosis  Malignant: 33 IDC, 7 ILC, 2 mixed IDC/ILC, 2 MuC, 1 DCIS, 1 MeC | 1, 2, 3, 7, 11, 12 | 50 |
| [49] Kuroki  2004 | 5 | 55 | 0, 1000 | 1.448±0.453 | 1.021±0.23 | <0.0002 | Benign: 1 FA, 1 mastopathy, 3 adenosis  Malignant: 47 IDC, 3 DCIS, 3 ILC, 2 special types | 1, 2, 3, 5, 6, 12 | 50 |
| [50] Lo  2009 | 11 | 20 | 0, 1000 | 1.47±0.30 | 1.01±0.25 | 0.0002 | Benign: 1 FA, 7 FCC, 1 fat necrosis, 1 inflammatory change Malignant: 10 IDC, 8 DCIS, 1 tubular carcinoma, 1 MeC | 1, 2, 3, 5, 6, 12 | 50 |
| [51] Park  2007 | 4 | 52 | 0, 1000 | 1.41±0.56 | 0.89±0.18 | <0.05 | Benign: 1 FA, 2 FCC, 1 epidermal inclusion cyst  Malignant: 43 IDC, 1 MuC, 1 mixed infiltrative and MuC, 7 DCIS | 1, 2, 3, 5, 6, 12 | 50 |
| [52] Partridge  2010 | 91 | 27 | 0, 600 | 1.71±0.43 | 1.32±0.23 | <0.001 | Benign: 12 FA, 7 pseudoangiomatous stromal hyperplasia, 9 atypical ductal hyperplasia, 6 fibrosis, 57 other  Malignant: 11 DCIS, 15 IDC, 5 ILC | 1, 2, 3, 5, 6, 9, 10, 12 | 67 |
| [53] Pereira  2009 | 26 | 26 | 0, 250, 500, 750, 1000 | All b-values: 1.50±0.34,  b 0 and 1000: 1.44±0.31 | All b-values: 0.92±0.26,  b 0 and 1000: 0.68±0.25 | <0.0001 (alle b-waarden combinaties) | Benign: 3 FA, 1 epidermoid cyst, 1 granulomatous intramammary lymph node, 1 PP, 20 not specified  Malignant: 19 IDC, 2 DCIS, 2 tubular carcinoma, 1 adenoid cystic carcinoma, 1 MuC, 1 MPh | 1, 2, 3, 12 | 33 |
| [54] Rubesova 2006 | 22 | 65 | 0, 200, 400, 600, 1000 | 1.51±0.068 | 0.95±0.027 | <0.001 | Benign: NA Malignant: 41 IDC, 13 ILC, 2 mixed IDC/ILC, 3 LCIS, 6 DCIS | 1, 2, 3, 5, 6, 12 | 50 |
| [55] Satake  2011 | 27 | 88 | 50, 800, 1500 | 1.1±0.34 | 0.89±0.28 | <0.05 | Benign: 11 PP, 6 FA, 1 benign phyllodes tumour, 1 atypical ductal hyperplasia, 3 ductal hyperplasia, 2 sclerosing adenoma, 3 fibrosis and microcysts  Malignant: 4 DCIS, 73 IDC, 5 MuC, 6 ILC | 1, 2, 3, 4, 5, 7, 9, 12 | 67 |
| [56] Sinha  2002 | 6 | 17 | 5 b-values from 0 to 289.7 | Benign:  2.01± 0.46 (Cyst: 2.65±0.30) | 1.60±0.36 | p<0.05 | Benign: NA (excl cyst)  Malignant: NA | 1, 2, 3, 5, 6 | 42 |
| [57] Sonmez  2011 | 20 | 25 | 0, 50, 200, 500, 1000 | 1.49±0.16 | 0.82±0.07 | NA | Benign: 15 FA, 5 other  Malignant: 19 IDC, 4 ILC, 2 apocrine carcinoma | 1, 2, 3, 5, 6, 7, 12 | 58 |
| [58] Stadlbauer 2009 | 27 | 9 | 0, 1000 | 1.59±0.37 | 0.99±0.26 | <0.001 | Benign: 12 FA, 5 cysts, 7 lymph nodes, 3 postoperative scars Malignant: IDC + DCIS (?) | 1, 2, 3, 5, 6 | 42 |
| [59] Tozaki  2009 | 48 | 63 | 500, 1500 | 1.19 | 0.73 | <0.001 | Benign: 25 FA, 12 PP, 6 benign proliferative disease, 2 ductal adenoma, 1 radial scar, 1 lymph node, 1 abscess  Malignant: 44 IDC, 10 DCIS, 2 MuC, 2 invasive micropapillary carcinoma, 2 apocrine carcinoma, 1 ILC, metaplastic carcinoma, 1 MPh | 1, 2, 3, 5, 6, 12 | 50 |
| [60] Woodhams 2005 | 24 | 167 | 0, 750, 1000 | 1.67±0.54 | 1.22±0.31 | ‘statistically significant difference’ | Benign: 2 FA, 8 FCC, 7 ductal hyperplasia, 6 PP, 3 BPh, 1 sclerosing adenosis, 1 atypical ductal hyperplasia, 1 granuloma  Malignant: 138 IDC, 27 DCIS | 1, 2, 3, 5, 6 | 42 |
| [61] Woodhams 2009 | 58 | 204 | 0, 1500 | 1.3±0.3 | 0.9±0.2 | ? | Benign: 19 FA, 26 FCC, 4 PP, 3 BPh, 6 other Malignant: 98 IDC, 12 ILC, 15 MC, 15 DCIS, 1 LCIS | 1, 2, 3, 5, 6, 8, 9, 12 | 67 |
| [62] Yili  2009 | 22 | 35 | 500, 1000 | 1.73±0.34 | 1.01±0.20 | 0.000 | Benign: 14 FA, 8 breast hyperplasia  Malignant: 31 IDC, 3 DCIS | 1, 2, 3, 5, 6, 12 | 50 |

Note: QUADAS: Quality Assessment of the Studies of Diagnostic Accuracy Included in Systematic Reviews, NA: not available, FA: fibroadenoma, FCC: fibrocystic change, PP: papilloma, BPh: benign phyllodes tumour, MPh: malignant phyllodes tumour IDC: invasive ductal carcinoma, ILC: invasive lobular carcinoma, DCIS: ductal carcinoma in situ, LCIS: lobular carcinoma in situ, MuC: mucinous carcinoma, MeC: medullary carcinoma

Table S6: Lung

| Study | N benign | N malignant | b-values  s/mm2 | ADC benign x10-3 mm­­­2/s | ADC malignant x10-3 mm­­­2/s | P-value | Tumour types | Modified QUADAS items with positive score | Quality score % |
| --- | --- | --- | --- | --- | --- | --- | --- | --- | --- |
| [63] Uto  2009 | 10 | 18 | 0, 1000 | 1.151±0.310 | 1.019±0.362 | 0.388 | Benign: 6 inflammatory lesion, 1 sarcoidosis, 1 fungal infection, 1 nontuberculous myobacterial infection Malignant: 11 adenocarcinoma, 3 small cell carcinoma, 2 non-small cell carcinoma, 1 squamous cell carcinoma, 1 large cell carcinoma | 1, 2, 3, 5, 6, 7, 12 | 58 |
| [64] Liu  2010 | 12 | 54 | 0, 500 | 1.648±0.416 | 1.256±0.320 | 0.001 | Benign: 3 tuberculoma, 2 bacterial pneumonia, 1 inflammatory nodule, 1 hamartoma, 1 sclerosing haemangioma, 1 inflammatory pseudotumour, 1 schwannoma, 1 sarcoidosis, 1 lung abscess  Malignant: 15 squamous cell carcinoma, 14 adenocarcinoma, 5 bronchioalveolar carcinoma, 9 small cell carcinoma, 2 adenosquamous carcinoma, 1 sarcomatoid carcinoma, 2 poorly differentiated carcinoma, 7 metastasis | 1, 2, 3, 12 | 33 |

Note: QUADAS: Quality Assessment of the Studies of Diagnostic Accuracy Included in Systematic Reviews

Table S7: Liver

| Study | N benign | N malignant | b-values  s/mm2 | ADC benign x10-3 mm­­­2/s | ADC malignant x10-3 mm­­­2/s | P-value | Tumour type | Modified QUADAS items with positive score | Quality score % |
| --- | --- | --- | --- | --- | --- | --- | --- | --- | --- |
| [65] Battal | 102 | 41 | 0, 800 | 1.94±0.61 | 0.86±0.13 | <0.001 | Benign: 63 hemangioma, 25 simple cyst, 2 hydatid cyst, 3 adenoma, 7 FNH, 2 abscess  Malignant: 35 metastasis, 4 HCC, 2 cholangiocellular carcinoma | 1, 2, 3, 7, 9, 12 | 50 |
| [66] Choi  2010 | 19 | 31 | 0, 200, 400, 800 | 2.63±0.78  (cysts: 3.15±0.26) | 1.13±0.35 | <0.05 | Benign: 7 hemangioma, 12 cyst  Malignant: 18 HCC, 12 metastasis, 1 cholangiocarcinoma | 1, 2, 3, 7 | 33 |
| [67] Coenegrachts 2009 | 39 | 67 | 0, 10, 150, 400 | 2.118 | 1.097 | <0.001 | Benign: 39 hemangioma  Malignant: 67 metastasis | 1, 2, 3, 6, 7, 9, 12 | 58 |
| [68] Demir  2007 | 24 | 17 | 0, 1000 | 2.57±0.26  (cysts: 3.05±0.26) | 0.86±0.11 | <0.01 | Benign: 14 hemangioma, 6 simple hepatic cyst, 1 hydatid cyst, 3 abcess  Malignant: 8 metastasis, 3 HCC, 1 hepatoblastoma, 4 cholangiocellular carcinoma, 1 gall bladder tumour | 1, 2, 3, 7, 12 | 42 |
| [69] Holzapfel 2009 | 98 | 87 | 50, 300, 600 | 2.36±0.62  (cysts: 2.61±0.57) | 1.09±0.30 | <0.0001 | Benign: 71 cyst, 18 hemangiomas, 6 FNH, 3 adenoma  Malignant: 76 metastasis, 11 HCC | 1, 2, 3, 6, 9, 12 | 50 |
| [70] Kim  1999 | 30 | 49 | 3, 57, 192, 408, 517, 705, 846 | 2.49±1.39  (cysts: 2.91±1.51) | 1.01±0.38 | <0.0024 | Benign: 21 cyst, 16 hemangioma, 1 angiomyolipoma  Malignant: 35 HCC, 18 metastasis, 1 intrahepatic cholangiocellular carcinoma | 1, 2, 3, 7, 12 | 42 |
| [71] Koike  2009 | 18 | 67 | 0, 1000 | Hemangioma: 1.84±0.37  Cyst: 2.61±0.45 | HCC: 1.31±0.28 metastasis: 1.11±0.22 | ? | Benign: 10 hemangioma, 8 cyst  Malignant: 29 HCC, 4 cholangiocarcinoma, 34 metastasis | 1, 2, 3, 7, 12 | 42 |
| [72] Miller  2010 | 323 | 219 | 0, 500 | 2.50±0.86 (all benign) | 1.52±0.55 | <0.05 | Benign: 95 cysts, 166 hemangioma, 43 FNH, 9 adenoma, 10 abscess  Malignant: 107 metastasis, 112 HCC | 1, 2, 3, 7, 9, 12 | 50 |
| [73] Onur  2012 | 26 | 69 | 100, 600, 1000 | 1.52±0.47 | 1.08±0.22 | <0.0001 | Benign: 14 FNH, 4 adenoma, 2 angiomyolipoma, 1 teratoma, 2 nodular regenerative hyperplasia, 3 alveolar echinococcose  Malignant: 28 HCC, 33 metastasis, 8 cholangiocarcinoma | 1, 2, 3, 7, 12 | 42 |
| [74] Papanikolaou 2010 | 14 | 13 | 0, 50, 500, 1000 | 2.86 | 1.307 | <0.0001 | Benign: 8 simple cyst, 4 hemangioma, 1 cystadenoma, 1 neurofibromatosis  Malignant: 12 metastasis, 1 HCC | 1, 2, 6, 12 | 33 |
| [75] Parikh  2008 | 75 | 136 | 0, 50, 500 | 2.19±0.67  (cysts: 2.54±0.67) | 1.39±0.38 | <0.001 | Benign: 37 cyst, 23 hemangioma, 5 adenoma, 5 abscess, 4 FNH, 1 posttransplantation intrahepatic hematoma  Malignant: 77 HCC, 59 metastatis | 1, 2, 3, 7, 9, 12 | 50 |
| [76] Sandrasegaran  2009 | 45 | 31 | 0, 50, 400 | Hemangioma: 1.568±0.541 cyst: 1.902±0.430  FNH: 1.301±0.819 | HCC: 1.076±0.327 | Solid benign (adenoma, FNH) vs malignant: no significant difference P=0.98 | Benign: 6 FNH, 3 adenoma, 5 cyst, 17 hemangioma  Malignant: 41 HCC, 4 metastasis | 1, 2, 3, 7, 9, 12 | 50 |
| [77] Taouli  2009 | 18 | 11 | 0, 500 | BH DWI: 2.21±0.60, PACE DWI 2.39±0.44 | BH DWI: 1.04±0.27, PACE DWI: 1.16±0.33 | BH DWI: p=0.082, PACE DWI: p=0.068 | Benign: 9 cyst, 8 hemangioma, 1 FNH  Malignant: 7 HCC, 4 metastasis | 1, 2, 3, 12 | 33 |
| [78] Taouli  2003 | 28 | 24 | 0, 500 | 2.45±0.96  (cyst: 3.63±0.56,  hemangioma: 2.95±0.67) | 1.08±0.50 | <0.01 | Benign: 12 FNH, 3 adenoma, 7 hemangioma, 6 cyst  Malignant: 15 metastasis, 9 HCC | 1, 2, 3, 7, 9, 11, 12 | 58 |

Note: QUADAS: Quality Assessment of the Studies of Diagnostic Accuracy Included in Systematic Reviews, HCC: Hepatocellular carcinoma, FNH: focal nodular hyperplasia

Table S8: Gallbladder

| Study | N benign | N malignant | b-values  s/mm2 | ADC benign x10-3 mm­­­2/s | ADC malignant x10-3 mm­­­2/s | P-value | Tumour types | Modified QUADAS items with positive score | Quality score % |
| --- | --- | --- | --- | --- | --- | --- | --- | --- | --- |
| [79] Sugita  2009 | 14 | 15 | 0, 1000 | 1.92±0.21 | 1,28±0.41 | <0.01 | Benign: 8 chronic cholecystitis, 3 adenomyomatosis, 3 gallbladder polyps Malignant: 15 gallbladder carcinoma | 1, 2, 3, 5, 6, 12 | 50 |

Note: QUADAS: Quality Assessment of the Studies of Diagnostic Accuracy Included in Systematic Reviews

Table S9: Pancreas

| Study | N benign | N malignant | b-values  s/mm2 | ADC benign x10-3 mm­­­2/s | ADC malignant x10-3 mm­­­2/s | P-value | Tumour types | Modified QUADAS items with positive score | Quality score % |
| --- | --- | --- | --- | --- | --- | --- | --- | --- | --- |
| [80] Fattahi 2009 | 14 | 10 | 0, 600 | 2.09±0.18 | 1.46±0.18 | <0.0001 | Benign: 14 MFP  Malignant: 10 DAC | 1, 2, 3, 7, 12 | 42 |
| [81] Huang  2011 | 14 | 37 | 0, 1000 | 1.35±0.14 | 1.03±0.15 | <0.01 | Benign: 14 MFP  Malignant: 37 PC | 1, 2, 3, 4, 5, 7 |  |
| [82] Kamisawa  2010 | 13 | 40 | 0, 50, 800 | 1.01±0.11 | 1.25±0.11 | <0.001 | Benign: autoimmune pancreatitis  Malignant: 40 PC | 1, 2, 3, 7, 12 | 42 |
| [83] Kartalis  2009 | 24 | 12 | 0, 500 | 2.57±1.17 | 1.40±0.30 | 0.0025 | Benign: NA  Malignant: NA | 1, 2, 3, 7, 9, 12 | 50 |
| [84] Klauss  2011 | 9 | 11 | 0, 800 | 1.27±0.10 | 1.18±0.19 | 0.116 | Benign: 9 MFP  Malignant: 18 ductal adenocarcinoma, 1 anaplastic carcinoma, 1 adenocarcinoma of uncertain origin | 1, 2, 3, 5, 7, 9, 12 | 58 |
| [85] Lee  2008 | 13 | 47 | 0, 500, 1000 | 1.04±0.18 | 1.23±0.18 | 0.004 | Benign: 13 MFP Malignant: 47 PC | 1, 2, 3, 12 | 33 |
| [86] Sandrasegaran  2011 | 66 | 4 | 50, 400, 800 | 2.42 | 1.98 | 0.06 | Benign: 28 IPMN, 9 MCN, 16 serous cystadenoma, 13 pseudocysts  Malignant: 4 ductal adenocarcinoma | 1, 2, 3, 7, 9, 12 | 50 |
| [87] Takeuchi 2008 | 4 | 20 | 0, 800 | 1.00±0.18 | 1.38±0.38 | <0.05 | Benign: 4 MFP  Malignant: 20 PC | 1, 2, 3, 9, 12 | 42 |
| [88] Yamashita 1998 | 15 | 15 | 30, 300 | pseudocysts: 3.2±1.3 | 2.7±0.9 | No significant difference | Benign: NA  Malignant: NA | 1, 2, 3, 12 | 33 |

Note: QUADAS: Quality Assessment of the Studies of Diagnostic Accuracy Included in Systematic Reviews, MFP: mass-forming pancreatitis, DAC: ductal adenocarcinoma, PC: pancreatic cancer (not specified), NA: not available

Table S10: Kidney

| Study | N benign | N malignant | b-values  s/mm2 | ADC benign x10-3 mm­­­2/s | ADC malignant x10-3 mm­­­2/s | P-value | Tumour types | Modified QUADAS items with positive score | Quality score % |
| --- | --- | --- | --- | --- | --- | --- | --- | --- | --- |
| [89] Abdel Razek | 9 | 45 | 0, 800 | 1.87±0.50 | 1.56±0.26 | 0.001 | Benign: 4 AML, 4 oncocytoma, 1 papillary adenoma  Malignant: 19 clear RCC, 6 papillary RCC, 8 chromophobe RCC, 2 unclassified RCC, 4 XP11 translational cancer, 2 collecting duct cancer, 2 Wilms tumour, 2 PNET | 1, 2, 3, 4, 5, 7, 9, 12 | 67 |
| [90] Doganay  2011 | 35 | 32 | 100, 600, 1000 | b-1000:  2.10±0.93 | b-1000:  1.66±0.51 | 0.02 | Benign: 12 AML, 2 textilloma, 18 cyst, 4 autosomal recessive polycystic kidney  Malignant: 22 RCC, 3 oncocitoma, 3 lymphoma, 3 translational cell carcinoma | 1, 2, 3, 7, 9, 12 | 50 |
| [91] Kilickesmez  2009 | 46 | 21 | 0, 500, 1000 | simple cysts: 2.94±0.20, hemorrhagic cysts: 1.71±0.37, hydronephrosis: 1.53±0.25, angiomyolipoma: 1.40±0.21 | RCC: 1.05±0.39, metastasis: 1.50±0.12 | ? | Benign: 17 simple cyst, 10 hemorrhagic cyst, 10 hydronephrosis, 9 angiomyolipoma  Malignant: 16 RCC, 5 metastasis | 1, 2, 3, 7, 12 | 42 |
| [92] Kim  2009 | 38 | 26 | 0, 400 | 2.50±0.53 | 1.75±0.57 | <0.0001 | Benign: 38 T1 hyperintense benign cyst  Malignant: 26 RCC | 1, 2, 3, 7, 9, 12 | 50 |
| [93] Sandrasegaran  2010 | 34 | 25 | 0, 400, 800 | 2.72 | 1.88±0.21 | <0.0001 | Benign: 31 cyst, 3 abscess  Malignant: 17 clear cell RCC, 5 papillary RCC, 1 chromophobe RCC, 2 transitional cell RCC | 1, 2, 3, 6, 7, 9, 12 | 58 |
| [94] Taouli  2009 | 81 | 28 | 0, 400, 800 | 2.23±0.87 (all benign lesions), 2.49±0.68 (benign lesions, excluding AMLs) | 1.41±0.61 | <0.0001 | Benign: 7 hemorrhagic cysts, 8 septated cyst, 2 multilocular cystic nephroma, 1 renal abscess, 10 AML, 6 oncocytoma  Malignant: 12 papillary RCC, 11 clear cell RCC, 1 chromophobe RCC, 1 adenocarcinoma, 3 unspecified | 2, 3, 7, 9, 10, 11, 12 | 58 |
| [95] Yoshikawa  2006 | 55 | 12 | 0, 600 | Angiomyolipoma 1.81±0.41, cyst 3.82±0.39, complicated cyst: 2.78±0.71 | RCC: 2.49±0.72 | Angiomyolipoma vs RCC: <0.0005, cyst vs RCC: <0.0001 | Benign: 42 cyst, 5 complicated cyst, 8 AML Malignant: 12 RCC | 1, 2, 3, 7, 12 | 42 |
| [96] Zhang  2008 | 11 | 26 | 0, 500, 1000 | benign cysts: mean 3.269±0.617 | tumours: 1.880±0.434 | <0.001 | Benign: 11 cyst  Malignant: 20 RCC, 1 angiosarcoma, 1 collecting duct carcinoma, 1 PNET, 2 oncocytoma, 1 angiomyolipoma | 1, 2, 3, 7, 9, 12 | 50 |

Note: QUADAS: Quality Assessment of the Studies of Diagnostic Accuracy Included in Systematic Reviews, AML: angiomyolipoma, RCC: renal cel carcinoma, PNET: primitive neuroectodermal tumour

Table S11: Adrenal gland

| Study | N benign | N malignant | b-values  s/mm2 | ADC benign x10-3 mm­­­2/s | ADC malignant x10-3 mm­­­2/s | P-value | Tumour types | Modified QUADAS items with positive score | Quality score % |
| --- | --- | --- | --- | --- | --- | --- | --- | --- | --- |
| [97] Miller  2010 | 141 | 19 | 0 or 50, 500 and 1000 | Median benign: 1.61 | Median malignant: 1.67 | No significant difference between benign and malignant | Benign: 118 adenoma, 9 myelolipoma, 9 cysts, 4 hemorrhage, 1 angiolipoma  Malignant: 11 metastasis, 4 cortical carcinoma, 3 pheochromocytoma, 1 neuroblastoma | 1, 2, 3, 9, 12 | 42 |
| [98] Sandrasegaran  2011 | 37 | 12 | 0 or 50, 400 or 500 and 800 | 1.35±0.64 | 0.88±0.29 | 0.02 | Benign: 36 nonfunctioning adenoma, 1 pheochromocytoma  Malignant: 6 metastasis from renal cell carcinoma, 3 metastasis from hepatocellular carcinoma, 2 pheochromocytoma, 1 adrenal cortical carcinoma | 1, 2, 3, 7, 9, 12 | 50 |
| [99] Song  2011 | 40 | 7 | 0, 800 | Benign pheochromocytomas: 1.15, lipid-poor adenomas: 1.07 | 0.92 | 0.04 (benign vs malignant) | Benign: 22 benign pheochromocytoma, 18 lipid-poor adenoma  Malignant: 7 malignant tumour | 1, 2, 3, 7, 12 | 42 |
| [100] Tsushima  2009 | 31 | 12 | 0, 1000 | adenomas: 1.09±0.29 | metastatic tumours: 0.85±0.26, pheochromocytomas: 1.59±0.34 | adenomas vs metastases: 0.14, adenoma vs pheochromocytomas: <0.05, pheochromocytoma vs metastases: <0.005 | Benign: 31 cortical adenoma Malignant: 7 metastasis, 5 pheochromocytoma | 1, 2, 3, 12 | 33 |

Note: QUADAS: Quality Assessment of the Studies of Diagnostic Accuracy Included in Systematic Reviews

Table S12: Uterus

| Study | N benign | N malignant | b-values  s/mm2 | ADC benign x10-3 mm­­­2/s | ADC malignant x10-3 mm­­­2/s | P-value | Tumour types | Modified QUADAS items with positive score | Quality score % |
| --- | --- | --- | --- | --- | --- | --- | --- | --- | --- |
| [101] Fujii  2008 | 12 | 13 | 0, 1000 | 1.44±0.34 | 0.98±0.19 | <0.01 | Benign: 8 submucosal leiomyoma, 4 endometrial polyp  Malignant: 11 endometrial carcinoma, 2 carcinosarcoma | 1, 2, 3, 5, 6, 7, 12 | 58 |
| [102] Inada  2009 | 44 | 23 | 0, 800 | leiomyoma: 1.35±0.24, adenomyosis: 1.21±0.19 | 0.97±0.19 | cancer vs leiomyoma: <0.0001, cancer vs adenomyosis: 0.0041 | Benign: 34 leiomyoma, 10 adenomyosis  Malignant: 23 endometrial adenocarcinoma | 1, 2, 3, 5, 6, 7, 12 | 58 |
| [103] Koc  2012 | 11 | 9 | 0, 600, 800, 1000 | 1.60±0.60 | 1.03±0.25 | 0.009 | Benign: 6 myoma uteri, 2 mole hydatiforme, 2 endometrial hyperplasia, 1 decidual remnant  Malignant: 9 endometrial carcinoma | 1, 2, 3, 5, 6, 7, 9, 12 | 67 |
| [104] Namimoto  2009 | 95 | 8 | 0, 1000 | 1.18±0.24 | 0.86±0.11 | <0.01 | Benign: 95 leiomyoma  Malignant: 8 sarcoma | 1, 2, 3, 9 | 33 |
| [105] Shen  2008 | 7 | 21 | 0, 500, 800, 1000 | 1.277±0.219 | 0.864±0.311 | 0.0058 | Benign: 4 endometrial hyperplasia, 3 endometrial polyp  Malignant: 21 endometrial adenocarcinoma | 1, 2, 3, 5, 6, 12 | 50 |
| [106] Takeuchi  2009 | 27 | 7 | 0, 800 | 1.51±0.33 (degenerated leiomyoma: 1.60, cellular leiomyomas: 1.18) | 0.76±0.26 | <0.01 | Benign: 6 cellular leiomyoma, 21 degenerated leiomyoma  Malignant: 2 undifferentiated sarcoma, 2 rhabdomyosarcoma, 1 leiomyosarcoma, 1 endometrial stromal sarcoma, 1 malignant lymphoma | 1, 2, 3, 5, 6, 9, 12 | 58 |
| [107] Tamai  2008 | 51 | 7 | 0, 500, 1000 | ordinary leiomyomas: 0.88±0.27, degenerated leiomyomas: 1.70±0.11, cellular leiomyomas: 1.19±0.18 | uterine sarcomas: 1.17±0.15 | ? | Benign: 43 ordinary leiomyoma, 6 degenerated leiomyoma, 2 cellular leiomyoma  Malignant: 7 uterine sarcoma | 1, 2, 3, 5, 6, 9, 12 | 58 |
| [108] Wang  2010 | 28 | 19 | 0, 1000 | 1.637±0.178 | 0.878±0.185 | <0.01 | Benign: 12 endometrial hyperplasia, 7 endometrial polyp  Malignant: 28 endometrial adenocarcinoma | 1, 2, 3, 4, 5, 7, 9, 12 | 67 |

Note: QUADAS: Quality Assessment of the Studies of Diagnostic Accuracy Included in Systematic Reviews

Table S13: Ovaries

| Study | N benign | N malignant | b-values  s/mm2 | ADC benign x10-3 mm­­­2/s | ADC malignant x10-3 mm­­­2/s | P-value | Tumour types | Modified QUADAS items with positive score | Quality score % |
| --- | --- | --- | --- | --- | --- | --- | --- | --- | --- |
| [109] Fujii  2008 | 81 | 42 | 0, 1000 | 1.47±0.42  (solid component) | 1.41±0.34 (solid component) | >0.05 (not significant) | Benign: 7 fibroma, 22 mucinous adenoma, 1 struma ovarii, 18 mature cystic teratoma, 7 serous adenoma, 24 endometrioma, 1 decidualized endometrioma, 1 polypoid endometrioma  Malignant: 19 SAC, 6 CCAC, 4 EAC, 1 dysgerminoma, 1 leiomyosarcoma, 4 metastatic tumour, 2 granulosa cell tumour, 2 mucinous borderline tumour, 1 endometrioid borderline tumour, 1 serous borderline tumour | 1, 2, 3, 5, 6, 7, 12 | 58 |
| [110] Katayama  2002 | 56 | 11 | 200, 400, 1000 | endometrial cysts: 1.24±0.46, dermoid cysts: 1.27±0.66, serous cystadenomas 1.64±0.14, mucinous cystadenomas: 1.61±0.61  (all cystic component) | 1.64±0.48 (cystic component) | >0.05 (not significant) | Benign: 10 dermoid cyst, 9 endometrial cyst, 1 serous cystadenoma,  Malignant: 4 mucinous cystadenoma including borderline malignancy, 1 endometrial adenocarcinoma, 3 clear cell adenocarcinoma, 3 serous adenocarcinoma | 1, 2, 3, 5, 6, 7 | 50 |
| [103] Koc  2012 | 20 | 11 | 0, 600, 800, 1000 | 2.26±0.82  (cystic and solid components combined) | 1.10±0.17 (cystic and solid components combined) | 0.001 | Benign: 10 serous cysts, 2 mass forming corpus albicans, 3 hemorrhagic cyst, 1 endometrioma, 2 mature cystic teratoma, 1 fibrothecoma, 1 mucinous cystadenoma  Malignant: 7 serous carcinoma 1 undifferentiated carcinoma, 1 dysgerminoma, 2 borderline mucinous tumour | 1, 2, 3, 5, 6, 7, 9, 12 | 67 |
| [111] Li  2011 | 46 | 85 | 0, 1000 | 2.58±0.27  (cystic component)  1.69±0.25  (solid component) | 2.44±0.33 (cystic component)  1.03±0.22  (solid component) | Not significant for cystic components,  <0.01 for solid components | Benign: 26 serous cystadenoma, 16 mucinous cystadenoma, 2 serous cystoadenofibromas, 1 Brenner tumour  Malignant: 33 serous cystadenocarcinoma, 12 borderline serous cystadenomas, 9 borderline mucinous cystadenoma, 3 endometroid adenocarcinoma, 2 CCAC, 1 undifferentiated adenocarcinoma | 1, 2, 3, 5, 7, 12 | 50 |
| [112] Nakayama  2005 | 107 | 24 | 0, 500, 1000 | 1.33±0.82 (cystic component) | 2.28±0.32 (cystic component) | <0.001 | Benign: 54 mature cystic teratoma, 35 endometrial cyst, 13 cystadenoma, 1 fibroma, 3 paraovarian cysts, 1 torsion of benign cyst  Malignant: 5 EAC, 4 adenocarcinoma, 4 serous papillary adenocarcinoma, 3 metastatic adenocarcinoma, 2 mucinous cystadenocarcinoma, 1 mucinous borderline malignancy, 2 clear-cell carcinoma, 2 immature teratoma, 1 squamous cell carcinoma | 1, 2, 3, 4, 5, 6, 7, 12 | 67 |
| [113] Takeuchi  2010 | 10 | 39 | 0, 800 | 1.38±0.30  (solid component) | 1.03±0.19  (solid component) | <0.001 | Benign: 2 cystadenofibroma, 3 thecoma, 4 fibroma, 1 struma ovarii  Malignant: 29 surface epithelial-stromal tumour, 3 metastasis, 1 carcinoid, 6 borderline malignant tumour | 1, 2, 3, 5, 7, 9, 12 | 58 |
| [114] Thomassin-Nagarra  2009 | 30 | 47 | 0, 500 | cystic component: 2.32±0.56, solid component: 1.15±0.55 | cystic component: 2.34±0.47, solid component: 1.14±0.28 | cystic components of malignant vs benign: p=0.82, solid component of malignant vs benign: p=0.89 | Benign:16 epithelial cystadenoma, 10 benign sex cord-stromal tumour, 2 tubo ovarian abscess, 1 hydrosalpinx, 1 hemorrhagic luteal cyst  Malignant: 30 cystadenocarcinoma, 8 borderline tumour, 3 malignant sex cord-stromal tumour, 4 metastasis, 2 tubal cancer | 1, 2, 3, 5, 6, 12 | 50 |

Note: QUADAS: Quality Assessment of the Studies of Diagnostic Accuracy Included in Systematic Reviews, SAC: serous adenocarcinoma, MAC: mucinous adenocarcinoma, CCAC: clear cell adenocarcinoma, EAC: endometrioid adenocarcinoma

Table S14: Soft-tissue

| Study | N benign | N malignant | b-values  s/mm2 | ADC benign x10-3 mm­­­2/s | ADC malignant x10-3 mm­­­2/s | P-value | Tumour types | Modified QUADAS items with positive score | Quality score % |
| --- | --- | --- | --- | --- | --- | --- | --- | --- | --- |
| [115] Einarsdóttir  2004 | 16 | 13 | 0, 600 | 1.8 | 1.7 | not significant | Benign: 5 desmoid, 3 schwannoma, 2 benign fibroblastic proliferations, 2 myxoma, 1 haemangioma, 1 tumourous calcinosis, 1 cystic, 1 ganglion  Malignant: sarcoma | 1, 2, 3, 12 | 33 |
| [116] Maeda  2007 | 18 | 26 | 5, 1000 | 1.50±0.64 | 1.45±0.59 | not significant | Benign: 7 Myxoid: 3 myxoma, 2 schwannoma, 1 neurofibroma, 1 spindle cell lipoma, 11 nonmyxoid: 5 epidermal cyst, 4 haemangioma, 1 pleomorphic lipoma, 1 desmoid  Malignant: 16 myxoid: 9 myxoid liposarcoma, 3 myxofibrosarcoma, 3 UHGPS, 1 extraskeletal myxoid chondrosarcoma, 10 nonmyxoid: 2 lymphoma, 2 synovial sarcoma, 2 extraskeletal Ewing sarcoma, 1 well-differentiated liposarcoma, 1 malignant peripheral nerve sheath tumour, 2 undifferentiated high grade pleomorphic sarcoma | 1, 2, 3, 5, 6, 7, 12 | 58 |
| [117] Nagata  2008 | 44 | 36 | 0, 1000 | 1.70±0.62 | 1.19±0.58 | not significant | Benign: 22 myxoid: 18 schwannoma, 4 neurofibroma, 22 nonmyxoid: 5 giant cell tumour of the tendon sheath, 4 angioleiomyoma, 4 calcifying epithelioma, 2 diffuse-type giant cell tumour, 1 haemangioma, 1 dermatofibroma, 1 soft tissue chondroma, 1 myofibroma, 1 glomus tumour, 1 spindle cell lipoma, 1 fibrolipoma  Malignant: 8 myxoid: 4 myxoid liposarcoma, 3 myxofibrosarcoma, 1 malignant fibrous histiocytoma, 28 nonmyxoid: 7 malignant lymphoma, 5 melanoma, 4 muscular metastasis, 4 pleomorphic malignant fibrous histiocytoma, 2 alveolar soft part sarcoma, 1 dedifferentiated liposarcoma, 1 synovial sarcoma, 1 leiomyosarcoma, 1 sarcomatoid carcinoma, 1 angiosarcoma, 1 malignant cylindroma | 1, 2, 3, 5, 6, 12 | 50 |
| [118] Oka  2008 | 6 | 31 | 0, 500, 1000 | 1.55±0.121 | 0.92±0.139 | <0.01 | Benign: chronic expanding haematomas  Malignant: 13 malignant fibrous histiocytoma, 7 malignant peripheral nerve sheath tumours, 3 leiomyosarcomas, 3 rhabdomyosarcomas, 1 synovial sarcoma, 1 extraskeletal osteosarcoma, 1 extraskeletal Ewing sarcoma, 1 extrarenal rhabdoid tumour | 1, 2, 3, 5, 6, 12 | 50 |
| [119] Oka  2011 | 8 | 74 | 0, 500, 1000 | 1.36±0.48 | 0.88±0.20 | <0.01 | Benign: 8 desmoid tumour  Malignant: 21 undifferentiated pleomorphic sarcoma, 13 fibrosarcoma, 12 malignant peripheral nerve sheath tumour, 9 leiomyosarcoma, 6 thabdomyosarcoma, 6 synovial sarcoma, 2 alveolar soft part sarcoma, 2 extraskeletal Ewing’s sarcoma, 3 others | 1, 2, 3, 5, 7, 9, 10, 12 | 67 |
| [120] Van Rijswijk  2002 | 12 | 11 | 0, 176, 351, 526, 701 | 1.78±0.24 | 1.30±0.22 | not significant P>0.05 | Benign: 2 schwannomas, 2 fibromatosis, 1 myositis ossificans, 1 complex synovial cyst, 1 vascular malformation, 1 pigmented villonodular synovitis, 1 ganglion, 1 myxoma, 1 haematoma, 1 leiomyoma  Malignant: 5 liposarcoma, 2 myxofibrosarcoma, 1 leiomyosarcoma, 1 synovial sarcoma, 1 sarcoma not otherwise specified, 1 soft-tissue lymphoma | 1, 2, 3, 5, 6, 12 | 50 |

Note: QUADAS: Quality Assessment of the Studies of Diagnostic Accuracy Included in Systematic Reviews, UHGPS: undifferentiated high-grade pleomorphic sarcoma
